# Inclusive health: modeling COVID-19 in correctional facilities and communities

**DOI:** 10.1186/s12889-022-13313-7

**Published:** 2022-05-16

**Authors:** Scott Greenhalgh, Ashley Provencher

**Affiliations:** 1 Department of Mathematics, Siena College, 515 Loudon Road, Loudonville, NY 12211 USA; 2 Economics Department, Siena College, 515 Loudon Road, Loudonville, NY 12211 USA

**Keywords:** COVID-19, Disease modeling, Inequalities, Health inequalities, Modelling

## Abstract

**Background:**

Mass incarceration, commonly associated with overcrowding and inadequate health resources for incarcerated people, creates a fertile environment for the spread of the coronavirus disease 2019 (COVID-19) in U.S. correctional facilities. The exact role that correctional facilities play in enhancing COVID-19 spread and enabling community re-emergence of COVID-19 is unknown.

**Methods:**

We constructed a novel stochastic model of COVID-19 transmission to estimate the impact of correctional facilities, specifically jails and state prisons, for enhancing disease transmission and enabling disease re-emergence in local communities. Using our model, we evaluated scenarios of testing and quarantining infected incarcerated people at 0.0, 0.5, and 1.0 times the rate that occurs for infected people in the community for population sizes of 5, 10, and 20 thousand people.

**Results:**

Our results illustrate testing and quarantining an incarcerated population of 800 would reduce the probability of a major outbreak in the local community. In addition, testing and quarantining an incarcerated population would prevent between 10 to 2640 incidences of COVID-19 per year, and annually save up to 2010 disability-adjusted life years, depending on community size.

**Conclusions:**

Managing COVID-19 in correctional facilities is essential to mitigate risks to community health, and thereby stresses the importance of improving the health standards of incarcerated people.

**Supplementary Information:**

The online version contains supplementary material available at 10.1186/s12889-022-13313-7.

## Background

Cramped and overpopulated, correctional facilities are ideal environments for viruses to spread. This was made clear with the ongoing rapid spread of the coronavirus disease 2019 (COVID-19) in U.S. jails and prisons. As jails and prisons are structurally designed for communal living to efficiently confine people, the rate of infection is 5.5 times higher in U.S. state and federal prisons than in the broader community [1]. Limited access to personal protective equipment, hand sanitizer, and even soap exacerbates spread across all people within the facility. In prisons, 383,754 incarcerated people and 104,278 correctional staff have been diagnosed with COVID-19 as of February 2021 [2].

Though the poor health of incarcerated people and their limited access to health care has been widely documented, disparities are viewed primarily as injustices rather than a call for public health. The physical structures, including tall walls and barbed wires, suggest that these places are separate from local communities. But employees, volunteers, and visitors regularly move between surrounding communities and carceral facilities. Jails and prisons are not insulated spaces, particularly when it comes to airborne viruses. Viruses can thrive in congregate settings and easily transmit across places.

In contrast with other group living quarters, correctional facilities enable disease persistence and re-emergence. The reason for this is that many of the people who are housed within a correctional facility do not stay there for a long time. In the United States, on average, a person is confined to jail for 25 days [3], a state prison for 2.6 years [4], or to federal prison for 4.5 years [5]. The duration of incarceration varies by facility given differences in the carceral population housed in each place. Most people incarcerated in jail are pre-trial or pre-sentence or have been sentenced to less than 1 year. These facilities are under the jurisdiction of local governments, such as towns or counties. People who have been convicted and sentenced to more than 1 year of incarceration usually are incarcerated in state or federal prisons, with state or federal jurisdiction determining the type of prison. Correctional facilities, particularly jails, thus serve as reservoirs that enable disease persistence because people continually enter them without prior exposure to the virus, which facilitates its spread. Likewise, regular exits from correctional facilities may result in the virus’ re-emergence within surrounding communities if its spread is unchecked.

Recent works illustrate the severe risks for incarcerated individuals and correctional facilities [1–6]. To highlight a few of these risks, a COVID-19 outbreak is predicted to infect 72% of incarcerated people within a facility in 90 days [7], and the presence of COVID-19 in as little as 8 incarceration facilities would likely overwhelm the capacity of local health centers [8]. One study, similar to our work here, models the relationship between carceral institutions and community spread of COVID-19 [4]. However, despite these findings, little is known about the level that correctional facilities contribute to the persistence of COVID-19 in the community or how the implementation of COVID-19 control measures might mitigate these risks. So, to inform on these issues, we constructed a new stochastic model of COVID-19 transmission to estimate the impact of correctional facilities for enhancing disease transmission and enabling disease re-emergence. We conclude with a discussion of potential strategies to prevent disease persistence and re-emergence between correctional facilities and their local communities.

## Methods

We constructed a novel stochastic model, specifically a continuous-time Markov chain (CTMC) [6], of COVID-19 transmission to estimate the impact of correctional facilities in enabling disease transmission and disease re-emergence (Web Figure 1). We calibrated our model to describe COVID-19 spread in communities with correctional facilities that house 800 incarcerated people and staff 420 correctional workers, based on the setup of the largest county correctional facilities in New York State [7]. For such a facility, we measured how testing and quarantining infected incarcerated people at 0.0, 0.5, 1.0 times the rate that occurs for the general population impacts the spread of COVID-19 within the correctional facility and among the local community. In addition, to reflect the different population densities of rural and urban communities near correctional facilities, we also considered small, medium, and large communities of 5000, 10,000, and 20,000 people, respectively, in addition to the average incarceration period of 25 days in jails [3] 2.6 years in state prisons [4], along with the effects of social distancing and vaccination (Web Appendix 1). For each scenario, we computed 10,000 stochastic realizations over 1 year using Gillespie’s algorithm.

### Mathematical model

In the stochastic model, we considered a population segregated as the community (C), incarcerated people (P), and correctional workers (W), where correctional workers are defined as civilian employees or volunteers who reside outside of the correctional facility. We further subdivided each section of the population based on COVID-19 infection status using subscripts to denote people being susceptible (S), latently infected (E), asymptomatic infected (A), symptomatic infected (I), recovered from infection (R), acquired immunity from vaccination (V), hospitalized due to infection (H), premature death due to infection (D), and quarantined (Q).

To account for the difference in transmission risks among community members, correctional workers, and incarcerated people, our model included different rates for acquiring COVID-19 infection. The rate susceptible individuals in the community acquire COVID-19 is given by the force of infection:

1 $${\lambda}_{CW}=\alpha (t)\frac{\beta_{CW}}{C_{tot}+{W}_{tot}}\left({C}_I+{C}_A+{W}_I+{W}_A\right)$$

where *β*_*CW*_ is the transmission rate of COVID-19 in the community, *C*_*tot*_ is the total size of the community, *W*_*tot*_ is the total size of the correctional workers, and *α*(*t*) is the impact of public health control measures, such as face masks and social distancing, on mitigating COVID-19 spread in the community. Similarly, the rate susceptible correctional workers acquire COVID-19 is given by the force of infection:

2 $${\lambda}_{CW P}=\alpha (t)\frac{\beta_{CW}}{C_{tot}+{W}_{tot}}\left({C}_I+{C}_A+{W}_I+{W}_A\right)+\frac{\beta_{WP}}{P_{tot}+{W}_{tot}}\left({P}_I+{P}_A+{W}_I+{W}_A\right)$$

where *β*_*WP*_ is the transmission rate of COVID-19 in the correctional workers, and *P*_*tot*_ is the total size of the incarceration population. Finally, the rate susceptible incarcerated people acquire COVID-19 is given by the force of infection:

$${\lambda}_{WP}=\frac{\beta_{WP}}{P_{tot}+{W}_{tot}}\left({W}_I+{W}_A+{P}_I+{P}_A\right).$$

To reflect the influence of social distancing efforts on the transmission of COVID-19 in the community, we considered distinct phases of 1) pre-social distancing and 2) social distancing, along with the introduction of vaccination. These phases are reflected in the rate new infections occur through the function

$$\alpha (t)=\omega (N)\left\{\begin{array}{cc}1& t<50\ days\\ {}0.35& t\ge 50\ days\end{array}\right.,$$

where $\omega (N)=\frac{N-5000}{15000}0.619+0.381$ accounts for the spatial effects of Urban and Rural areas [8], in addition to the inclusion of the vaccination rate

$$\nu (t)=\left\{\begin{array}{cc}0& t<273\ da ys\\ {}0.00155& t\ge 273\ da ys\end{array}\right.\ da{y}^{-1}$$

Further details and additional model parameters based on [9–26], including the COVID-19 latent period, infectious period, the proportion of COVID-19 asymptomatic infections, and basic reproductive numbers of COVID-19 in the correctional facility and local community, are presented in Web Appendices 1–3, and Web Fig. 2.

### Transition probabilities

For our stochastic model, we used transition probabilities to determine the evolution between states. For the ease of presentation, we defined all states as

$$X=\left({C}_S,{C}_E,{C}_A,{C}_I,{C}_H,{C}_V,{C}_Q,{C}_D,{W}_S,{W}_E,{W}_A,{W}_I,{W}_H,{W}_V,{W}_Q,{W}_D,{P}_S,{P}_E,{P}_A,{P}_I,{P}_H,{P}_V,{P}_Q,{P}_D\right),$$

and let {*X*(*t*) : *t* ∈ *T*} be a random variable that represents all the state of the system at time *t* ∈ *T* = [0, ∞). Thus, assuming the Markov property, and given a sequence *t*_0_, *t*_1_, …, *t*_*n*_, *s*, *t* so that *t*_0_ < *t*_1_ < … < *t*_*n*_ < *s* < *t*, the transition probability from the *i*^*th*^ state to the *j*^*th*^ state for {*X*(*t*) : *t* ∈ *T*} is given by

$$P\left(X(t)=j|X(s)=i\right)={p}_{j\leftarrow i}\ \left(t-s\right).$$

As such, *X*(*s*) remains in its current state for *Δt* = *t* − *s* units of time before transitioning to *X*(*t*) with transition probability *p*_*j* ← *i*_ (*t* − *s*) = *p*_*j* ← *i*_ (Δ*t*). Further details of the transition probabilities between each state are available in Web Tables 1–4.

### Numerical simulation

To evaluate each intervention scenario, we implemented our model in the software platform R, and simulated stochastic realizations with Gillespie’s algorithm [6] using the computer cluster in Siena College’s High Performance Computing Center. The High Performance Computing Center consists of a total of 240 processing cores over 21 nodes, where each node has 500 gigabytes of local storage, 32 gigabytes of RAM, 32 gigabytes of RAM, and 2 E5–2600 Intel Xeon processors operating at 2.3 GHz.

### Intervention scenarios

To inform on the effect of identifying COVID-19 infected incarcerated people on transmission, we considered scenarios where the testing and quarantining rate of incarcerated people was 0, 0.5, and 1.0 times the rate used in the community *θ*_*C*_ (Web Table 5). We considered these testing and quarantining rates for total population sizes of 5000, 10,000, and 20,000 people, where the rate of incarceration and the average duration of incarceration reflected those occurring in jails and state prisons.

### Health outcomes

To determine the direct and indirect benefits of identifying and quarantining COVID-19 infected people on death and disability due to COVID-19, we measured health outcomes in incidence averted and disability-adjusted life years (DALYs) [27]. We considered annual incidence as the total number of symptomatic and asymptomatic COVID-19 infected individuals, and calculated time-discounted DALYs lost to COVID-19 [27], at the standard discount rate (Web Table 5), over 1 year. We determined the net DALYs saved by subtracting the total DALYs lost under no testing and quarantining from scenarios that consider identifying and quarantining COVID-19 infected incarcerated people. Disability weights for the DALY calculation and the proportion of people that endure each severity of COVID-19 were obtained from the literature [28–30]. In addition to calculating DALYs saved, we also estimated the hospitalizations and deaths averted through testing and quarantining incarcerated individuals.

### Community re-emergence

To provide insight into the potential role of correctional facilities in enabling COVID-19 reemergence, we estimated the likelihood of major outbreaks of COVID-19 in both correctional facilities and the community, in addition to the likelihood a correctional facility re-introduces COVID-19 into the community. Specifically, the probability of a major outbreak [31] within the correctional facility was estimated by $1-\frac{1}{R_0^P}$, and in the community with $1-\frac{1}{R_0^C}$, where ${R}_0^P$ and ${R}_0^C$ are the basic reproductive number of COVID-19 in these locations (Web Appendix 3), for total population sizes of 5000, 10,000, and 20,000 people. In addition, to provide insight into the risk of transmission from the correctional facility to the community, we determined the proportion of 10,000 simulations where a single infection in the correctional facility would lead to a transmission event in the community.

## Results

**Fig. 1:**
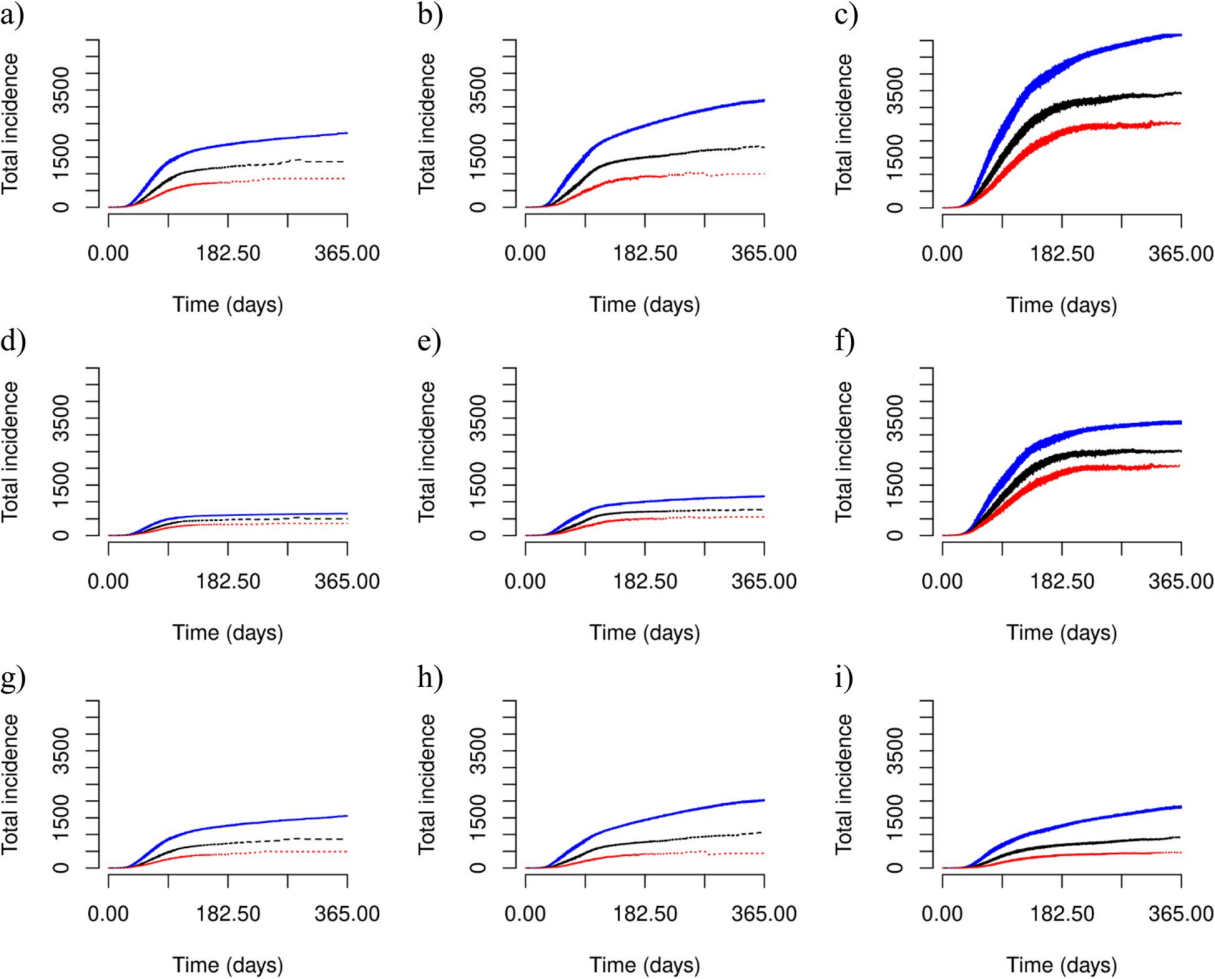
Cumulative COVID-19 incidence for an average duration of incarceration of 25 days. Predicted COVID-19 incidence in **a**-**c** the entire population, **d**-**f** the community (middle), and **g**)-**i**) in the correctional facility (bottom). Considered population sizes are **a**, **d** and **g** 5000 people, **b**, **e** and **h** 10,000 people, and **c**, **f** and **i** 20,000 people. Predicted COVID-19 incidence is illustrated for no intervention in correctional facilities (solid blue line), an intervention where testing and quarantining occur at 0.5 times the rate of the general population (dashed black line), and 1.0 times the rate of the general population (dotted red line) where the average duration of incarceration is 25 days

**Fig. 2:**
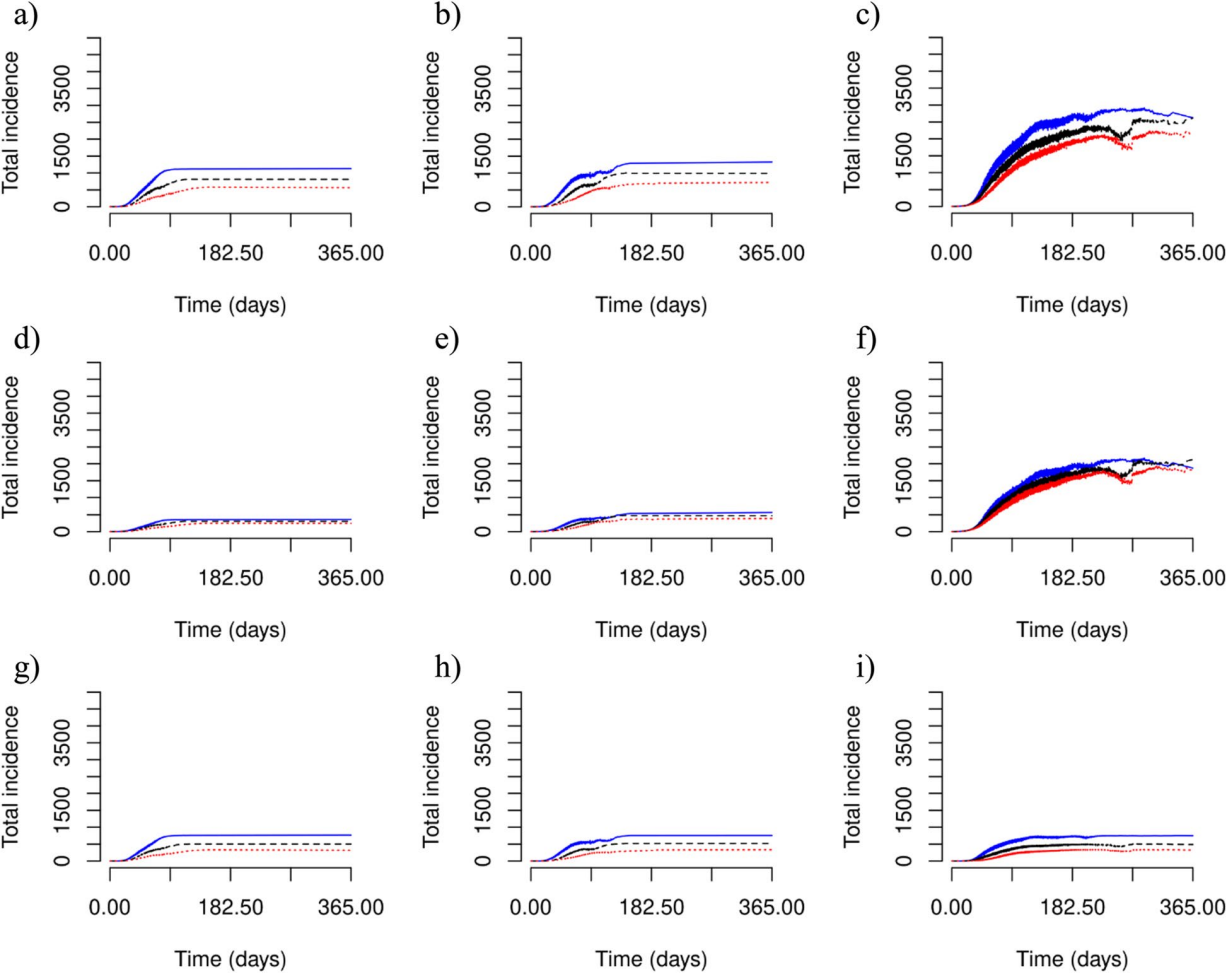
Cumulative COVID-19 incidence for an average duration of incarceration of 2.6 years. Predicted COVID-19 incidence in **a**-**c** the entire population, **d**-**f** the community (middle), and **g**-**i** in the correctional facility (bottom). Considered population sizes are **a**, **d** and **g** 5000 people, **b**, **e** and **h** 10,000 people, and **c**, **f** and **i** 20,000 people. Predicted COVID-19 incidence is illustrated for no intervention in correctional facilities (solid blue line), an intervention where testing and quarantining occur at 0.5 times the rate of the general population (dashed black line), and 1.0 times the rate of the general population (dotted red line) where the average duration of incarceration is 2.6 years

To inform on the potential role that correctional facilities play in COVID-19 transmission and the effect of testing and quarantining infected incarcerated people, we simulated transmission among incarcerated people, correctional workers, and people in the community. Our findings show that testing and quarantining infected incarcerated people substantially reduced COVID-19 incidence and saved DALYs (Figs. 1-2, Table 1-2, Web Figures. 4–11). Our findings also illustrate that smaller community sizes receive a greater reduction in COVID-19 incidence from testing and quarantining infected incarcerated people, a smaller risk of major COVID-19 outbreaks (Web Figure 3), in addition to a greater decrease in the risk for COVID-19 re-emergence.

**Table 1:** Simulation results for an average duration of incarceration of 25 days. Base annual COVID-19 incidence, annual incidence averted/1000, and annual DALYs saved/1000 people^a^

	*N* = 5000	*N* = 10000	*N* = 20000
No intervention (*θ*_*p*_ = 0.0 *θ*_*C*_)			
Baseline annual incidence (1000s)	2.21	3.19	5.18
Baseline annual incidence for community members (1000’s)	0.66	1.16	3.36
Baseline annual incidence for incarcerated people (1000’s)	1.56	2.02	1.82
Baseline total DALYs per 1000 people	2.25	2.69	5.87
Baseline hospitalizations per 1000 people	0.1	0.14	0.2
Baseline deaths per 1000 people	0.03	0.04	0.06
50% quarantine rate (*θ*_*P*_ = 0.5 *θ*_*C*_)			
Annual incidence averted (1000’s)			
Total population	0.85	1.40	1.75
Community	0.15	0.40	0.85
Incarcerated population	0.70	1.00	0.90
Total DALYs saved per 1000 people	0.83	1.14	4.77
Hospitalizations averted per 1000 people	0.04	0.07	0.08
Deaths averted per 1000 people	0.012	0.02	0.03
100% quarantine rate (*θ*_*P*_ = 1.0*θ*_*C*_)			
Annual incidence averted (1000’s)			
Total population	1.36	2.19	2.64
Community	0.29	0.61	1.30
Incarcerated population	1.07	1.59	1.35
Total DALYs saved per 1000 people	1.54	1.65	2.01
Hospitalizations averted per 1000 people	0.07	0.11	0.12
Deaths averted per 1000 people	0.03	0.04	0.04

^a^Annual incidence averted/1000, and annual DALYs saved/1000 people are calculated as the difference between the baseline and intervention scenario

**Table 2:** Simulation results for an average duration of incarceration of 2.6 years. Base annual COVID-19 incidence, annual incidence averted/1000, and annual DALYs saved/1000 people*

	*N* = 5000	*N* = 10000	*N* = 20000
No intervention (*θ*_*P*_ = 0.0*θ*_*C*_)			
Baseline annual incidence (1000s)	1.13	1.33	2.62
Baseline annual incidence for community (1000’s)	0.36	0.57	1.87
Baseline annual incidence for incarcerated people (1000’s)	0.77	0.76	0.75
Baseline total DALYs per 1000 people	0.58	0.81	4.32
Baseline hospitalizations per 1000 people	0.06	0.06	0.11
Baseline deaths per 1000 people	0.01	0.01	0.03
50% quarantine rate (*θ*_*P*_ = 0.5*θ*_*C*_)			
Annual incidence averted (1000’s)			
Total population	0.32	0.33	0.01
Community	0.06	0.10	0.25
Incarcerated population	0.26	0.24	0.26
Total DALYs saved per 1000 people	0.21	0.32	0.35
Hospitalizations averted per 1000 people	0.03	0.02	0.01
Deaths averted per 1000 people	0.009	0.001	0.006
100% quarantine rate (*θ*_*P*_ = 1.0*θ*_*C*_)			
Annual incidence averted (1000’s)			
Total population	0.56	0.60	0.47
Community	0.12	0.10	0.25
Incarcerated population	0.45	0.42	0.43
Total DALYs saved per 1000 people	0.25	0.25	0.53
Hospitalizations averted per 1000 people	0.04	0.04	0.03
Deaths averted per 1000 people	0.007	0.003	0.005

^*^Annual incidence averted/1000, and annual DALYs saved/1000 people are calculated as the difference between the baseline and intervention scenario

**Fig. 3:**
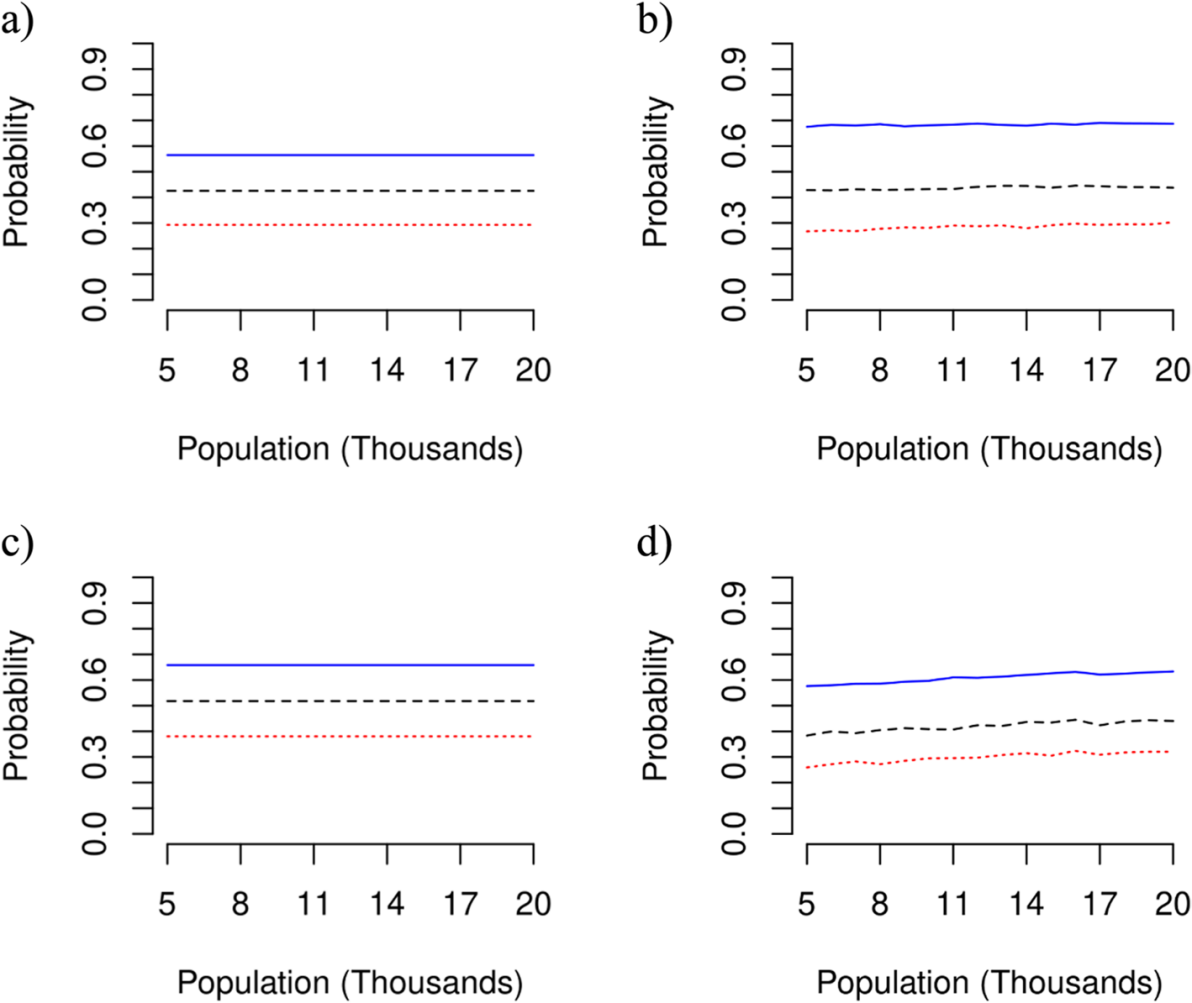
Probability of major outbreaks and incarceration-to-community transmission. The probability that a single COVID-19 infected incarcerated person results in (**a**) an outbreak in a jail, (**b**) a community transmission event from the jail, (**c**) an outbreak in a state prison, or (**d**) a community transmission even from the state prison, when there is no quarantining and testing of incarcerated people (solid blue line), when testing occurs at 0.5 times the community rate (dashed black line), or when testing occurs at the same rate as the community (dotted red line)

A single infected incarcerated person in a jail is more likely to lead to a community transmission event than cause a major COVID-19 outbreak within their correctional facility (Fig. 3a-d). Specifically, the probability that a single infected incarcerated person leads to a major outbreak of COVID-19 within a jail is 0.29, 0.43, or 0.56 (Fig. 3a). Similarly, the probability a single infected incarcerated person in a jail causes a community transmission event is approximately 0.29 0.42, and 0.69 (Fig. 3b), depending on whether quarantining and testing occurs at 1.0, 0.5, or 0.0 times the community rate, respectively.

We found that a single infected incarcerated person in a state prison had a lower risk to cause an outbreak in the correctional facility, with probabilities of 0.30, 0.42, and 0.6, in comparison to their counterparts in jails (Fig. 3a, c). We also found that the risk of a community transmission event from a state prison can be reduce by approximately 50% through quarantine and testing (Fig. 3d).

Upon the successful transmission of COVID-19 to outside the correctional facility, we found the probability of a major outbreak of COVID-19 in the community increased with the population (Web Appendix 3, Web Figure 3). For a population size of 20,000 people, testing and quarantining had a negligible impact on this probability, with the probability of a major outbreak for all scenarios of 0.67 approximately (Web Appendix 3, Web Figure 3). The probability of a major outbreak decreased for smaller community sizes, reaching a value of 0.09 for a population size of 5000 people (Web Appendix 3, Web Figure 3).

When considering a total population size of 5000 people that feature a nearby jail, we predict 2.21 annual incidents of COVID-19 per 1000 people annually, with 0.66 of these incidents occurring in the community, and 1.56 occurring within the correctional facility (Table 1). Annually, 85 and 136 incidents of COVID-19 can be averted when correctional facilities test and quarantine at 0.5 and 1.0 times the community rate, respectively (Table 1). Our results also illustrate the community benefits nearly as much as the incarcerated people from their testing and quarantining, concerning the reduction in COVID-19 incidence (Table 2), and annually saves 0.83 to 1.54 DALYs per 1000 people depending on the testing and quarantine scenario.

For a total population size of 10,000 people, COVID-19 incidence increased to 3.19 annual incidents per 1000 people, with 1.16 and 2.02 annual incidents per 1000 people occurring within the community and correctional facility, respectively (Table 1). Through testing and quarantining infected incarcerated people at 0.5 and 1.0 times the community rate, 1400 and 2190 annual incidents of COVID-19 can be averted, respectively, with nearly half of the averted incidents occurring in the community (Table 1). In addition, testing and quarantining incarcerated people at 0.5 the rate of the general public annually saves 1.14 DALYs per 1000 people, with this number increasing to 1.65 DALYs per 1000 people when testing and quarantining occurred at the same rate.

With a total population size of 20,000 people, COVID-19 incidence occurred at 5.18 annual incidents per 1000 people. Of these incidents, 3.36 and 1.82 incidents per 1000 people occur in the community, and the correctional facility, respectively (Table 1). By testing and quarantining infected incarcerated people at 0.5 and 1.0 times the community rate, we found that 1750 and 2640 annual incidents of COVID-19 can be averted (Table 1). Furthermore, from these total incidents averted, the majority occurs in the community with 850 to 1300 annual incidents of COVID-19 being averted, depending on the testing and quarantining rate. In addition, testing and quarantining incarcerated people at 0.5 the rate of the general public annually saves 2.01 DALYs per 1000 people, with this number increasing to 4.77 DALYs per 1000 people when testing and quarantining occurred at the same rate.

For COVID-19 transmission in state prisons, our model predicts 1.13, 1.33, and 2.62 annual incidents per 1000 people for total population sizes of 5000, 10,000, and 20,000 people, respectively. Through testing and quarantining at state prisons, these numbers can be reduced by 320 to 560, 330 to 600, and 10 to 470 incidences, respectively, depending on whether testing and quarantining incarcerated people occurs at 0.5 or 1.0 times the rate of the local population. To elaborate, if testing and quarantining incarcerated people occurs at 0.5 or 1.0 times the rate of the local population, then 60 to 250 incidences of COVID-19 would be averted in the community, and 240 to 450 incidences of COVID-19 would be averted in the state prison, depending on total population size (Table 2).

## Discussion

The analysis of our model of COVID-19 transmission between correctional facilities and local communities illustrates that testing and quarantining incarcerated people substantially reduces the health burden of COVID-19. Specifically, our model’s predictions illustrate that testing and quarantining incarcerated people reduces COVID-19 incidence in both correctional facilities and communities, reduces the likelihood of outbreaks in correctional facilities by up to 17%, and annually saves up to 4.77 DALYs per 1000 people.

Our work highlights a critical public health challenge: COVID-19 persists within correctional facilities and these facilities are likely to reintroduce the virus into local communities. At the forefront of what enables this public health challenge is that correctional facilities offer a reservoir of susceptible people that constantly changes given their short duration of incarceration. Indeed, an increasing number of empirical studies find a strong correlation between carceral institutions and community spread [2, 3, 5, 6]. Our model provides an explanation for causation.

While jails and state prisons have different average durations of incarceration, neither are closed systems that operate exclusively outside of local communities. Communities experiencing an outbreak of COVID-19 will likely lead to an outbreak within correctional facilities and vice versa. Highlighting this connection is the experience of Cook County, Illinois where nearly 16% of COVID-19 incidents were traced to the local jail [32]. Indeed, our simulations corroborate that the fallout from an outbreak within a correctional facility is dire for everyone. Fortunately, according to our results, testing and quarantining incarcerated individuals will substantially reduce the health burden of COVID-19 in both correctional facilities and communities. Specifically, testing and quarantining incarcerated people reduce the risks for major COVID-19 outbreaks and cross-transmission, causes a reduction in incidence in both correctional facilities and nearby communities, and saves DALYs. Together, these reduced risks and reductions provide strong motivation for the adoption of a policy that explicitly includes the health of incarcerated people when addressing community health.

Although our work illustrates a health benefit for testing and quarantining incarcerated individuals, a single policy is not sufficient to prevent outbreaks across all correctional facilities and communities. To elaborate, in the early days of the pandemic, many policymakers quickly adopted quarantine and early release policies to achieve greater social distancing within correctional facilities [33]. The populations of jails and prisons declined by 20 and 5%, respectively [34]. Of course, these policies are more difficult to enact in some facilities than others, which stresses that adopting one policy is not likely the most effective strategy to reduce virus spread. Furthermore, both the environment within the correctional facility and the community are important when determining COVID-19 mitigation and prevention strategies. For instance, our results illustrate that in communities with relatively small populations, the incidence of new cases stands to decline greatly if the correctional facility were able to quarantine incarcerated people or test incarcerated people at least at the same rate as people in the community. In contrast, while testing and quarantining reduce COVID-19 incidence in larger populations, it is less effective for curtailing the outbreak. This finding for larger facilities and communities suggests policies that reduce the number of incarcerated people, such as early release, are needed to diminish correctional facilities’ capacity to act as superspreading environments [6]. Furthermore, if early release policies are implemented, adequate post-release services should be robust and available to mitigate COVID-19 transmission risks [35].

Reducing the number of incarcerated people is one policy to mitigate the superspreading potential of incarceration facilities, but it is not the only one. With the development of COVID-19 vaccines, advocates and health policy researchers have called on policymakers to make vaccines available to incarcerated people during the earliest phases of distribution [36]. While this policy would likely mitigate the superspreading potential of correctional facilities, few states are adopting it, and most exclude incarcerated people [37]. Such actions, according to our findings, illustrate a lost opportunity to maximize health and safety, and suggest a more inclusive approach to COVID-19 vaccination would benefit everyone.

Policies aiming to reduce outbreaks within correctional facilities and local communities should be health-informed. Social distancing practices such as changes to housing or severe lockdowns within cells may mitigate spread within facilities, but will likely harm the mental well-being of incarcerated people [38, 39]. In contrast, issuing telephone cards for incarcerated people to stay in contact with family could improve mental well-being [40]. Other policies that improve sanitation, including access to disinfectants and personal protective equipment [40], or improve access to quality healthcare for incarcerated people, such as greater use of telemedicine [41], mitigate virus spread in correctional settings.

Findings from this study are limited in a few ways. First, data on the contacts between people in correctional facilities and the community are limited, although such limitations typically do not impede the widespread use of stochastic models in the study of disease transmission. We also did not account for the declining number of people in correctional facilities prior to the pandemic [42], nor the myriad of decarceration policies that occurred once the pandemic was underway, or the various subgroups of correctional workers, which include clergy, medical staff, and police officers. Furthermore, with regards to the policies of testing and quarantining of infectious individuals, our model assumed that these occur simultaneously, and did not account for the fact that their separate combination, through actions such as contact tracing and targeting at-risk persons, would likely save even more lives and mitigate disease spread further. Similarly, with regards to mitigating disease spread, our model assumed a standard population density in a correctional facility, though not all correctional facilities have the same layout, particularly as it relates to housing for incarcerated people. While dormitories and cells are the most common types of housing units, the availability and use of these spaces can vary considerably across facilities. Others [43, 44] have found evidence that the type of facility housing matters, and that people housed in dormitories are more susceptible to contracting the virus. As such, future models would do well to incorporate information on the varied population densities within correctional housing spaces to better understand viral spread and more accurately capture the potential heterogeneity in transmission.

There are several potentially fruitful future directions of our work. For instance, our model with modest adjustments could be adapted to other congregate settings, such as college dormitories [45, 46]. In addition, the inclusion of age-structure in the model would likely provide stronger estimates on COVID-19 related hospitalization and mortality rates in correctional facilities, and thereby provide stronger evidence for the design of optimal health policies. In a similar vein, future models should also consider the disproportionate impact of the pandemic on minorities [47, 48]. To elaborate, Blacks and Hispanics are overrepresented by 5.6 and 3.0 times more than White adults [42] in U.S. correctional facilities, which contributes to disparities of these groups in COVID-19 testing, cases, and deaths [3, 6]. As such, while our results provide a uniform estimate on the benefits of quarantining and testing for these groups, future research is urgently needed that investigates the intersection of race, health, and criminal justice involvement to better understand how criminal justice policy and practice may exacerbate health disparities.

## Conclusions

The health of incarcerated people likely has a substantial impact on the risk and magnitude of COVID-19 outbreaks in communities. Our findings illustrate that routine testing and quarantining of incarcerated people carries a dual benefit for correctional facilities and their local communities. Thus, our work suggests that to maximize public health officials’ ability to combat the ongoing COVID-19 pandemic, incarcerated people’s well-being should be included in the design and implementation of health policies.

## Supplementary Information

**Additional file 1.**

## Data Availability

All data generated or analyzed during this study are included in this published article (and its Supplementary Information files).

## Abbreviations

COVID-19: Coronavirus disease 2019
DALY: Disability-adjusted life years
CTMC: Continuous-time Markov chain
