# Supplementary material for: Inclusive health: modeling COVID-19 in correctional facilities and communities
Source: BMC Public Health. 2022 May 16;22:982. doi: 10.1186/s12889-022-13313-7 (PMC9108375; doi:10.1186/s12889-022-13313-7)
Supplement: Supplementary file 1 — Additional file 1. [file 12889_2022_13313_MOESM1_ESM.docx]

Web Material

INCLUSIVE HEALTH: MODELING COVID-19

IN CORRECTIONAL FACILITIES AND COMMUNITIES

**Table of Contents**

Web Appendix 1: COVID-19 Parameters

Web Appendix 2: Incarceration and Release Rates

Web Appendix 3: The Reproductive Numbers and the Probability of a Major Outbreak

Web Figure 1: Compartmental Diagram for COVID-19 Infection and Incarceration

Web Figure 2. Basic Reproductive Number for the Local Community.

Web Figure 3. Probability of a Major Outbreak in the Local Community.

Web Table 1: Transition Probabilities for COVID-19 Infection of Incarcerated Individuals

Web Table 2: Transition Probabilities for COVID-19 Infection of Incarcerated Workers

Web Table 3: Transition Probabilities for COVID-19 Infection of the Local Community Members

Web Table 4: Transition Probabilities for the Incarceration of Local Community Members and the Release of Incarcerated Individuals

Web Table 5: Parameter Names, Values, and Sources

Web Figure 4: Asymptomatic COVID-19 infections for communities with correctional facilities with an average duration of incarceration of 25 days

Web Figure 5. Symptomatic COVID-19 infections for communities with correctional facilities with an average duration of incarceration of 25 days

Web Figure 6. Hospitalized COVID-19 infections for communities with correctional facilities with an average duration of incarceration of 25 days

Web Figure 7. Deaths due to COVID-19 infections for communities with correctional facilities with an average duration of incarceration of 25 days.

Web Figure 8: Asymptomatic COVID-19 infections for communities with correctional facilities with an average duration of incarceration of 2.6 years

Web Figure 9. Symptomatic COVID-19 infections for communities with correctional facilities with an average duration of incarceration of 2.6 years

Web Figure 10. Hospitalized COVID-19 infections for communities with correctional facilities with an average duration of incarceration of 2.6 years

Web Figure 11. Deaths due to COVID-19 infections for communities with correctional facilities with an average duration of incarceration of 2.6 years

**Web Appendix 1: COVID-19 Parameters**

Model population sizes for the number of incarcerated people and correctional workers were determined using publicly available data on the capacity and annual occupancy of correctional facilities [[7]](https://paperpile.com/c/WghqcR/zOTV). The model also uses estimates of the duration of infection and the latent period of COVID-19 from the literature [[32, 33]](https://paperpile.com/c/WghqcR/JxI0+SMXD). In addition, the impact of pre- and post-social distancing [[34]](https://paperpile.com/c/WghqcR/iKcL) on COVID-19 transmission in the community was incorporated into model transmission rates. We also determined the rate of testing and quarantining of infected people from the community using available data on positive COVID-19 tests [[37]](https://paperpile.com/c/WghqcR/y9LU).

**The COVID-19 transmission rate and social distancing function.** The transmission rate of COVID-19 for our stochastic model is obtained from the literature [[37]](https://paperpile.com/c/WghqcR/y9LU) $\beta_{CW}\approx0.865/day$ without the use of social distancing or other interventions. To account for the effects of social distancing, we multiply $\beta_{CW}$ by the social distancing function $\alpha(t),$ given by

$$\alpha\left( t \right)=\omega\left( N \right)\left\{ \begin{matrix} 1 & t<50 days \\ 0.35 & t\geq50 days \end{matrix} \right..$$

Based on studies that examine the impact of diagnostic testing practices on COVID-19 transmission in the United States [[34]](https://paperpile.com/c/WghqcR/iKcL) [[39]](https://paperpile.com/c/WghqcR/iKcL), where $\omega\left( N \right)=\frac{N-5000}{15000}0.619+0.381$ accounts for the spatial effects of Urban and Rural areas [[8]](https://paperpile.com/c/WghqcR/Mg3E).

Note, we assume $\beta_{WP}\approx1.0383$ is not affected by social distancing due to the potential difficulties of implementing any such practice in correctional facilities [[38]](https://paperpile.com/c/WghqcR/zUVJ) (Web Figure 2).

**COVID-19 testing and quarantine rate.** We determine the testing and quarantining rate of the general community using available estimates on COVID-19 test rates [[37]](https://paperpile.com/c/WghqcR/y9LU). Specifically, we consider that $\theta_{C}=\theta_{W}\approx0.116/day.$

**COVID-19 vaccination rate.** We base the COVID-19 vaccination rates on estimates from the literature [[41]](https://paperpile.com/c/WghqcR/WAPN). Furthermore, we account for the delay in the distributing the vaccine based on the time from the NYS PAUSE declaration [[42]](https://paperpile.com/c/WghqcR/8MIs) to the time COVID-19 vaccines were given emergency use authorization [[43]](https://paperpile.com/c/WghqcR/upTt) of approximately 273 days later. Altogether, we have that

$$\nu\left( t \right)=\left\{ \begin{matrix} 0 & t<273 days \\ 0.00155 & t\geq273 days \end{matrix} \right. day^{-1}.$$

**Web Appendix 2: Incarceration and Release Rates**

To determine the rate of incarceration, $\rho_{in}$, we consider an correctional facility with a capacity of 800 people and assume that a person is confined for an average duration of $1/\rho_{out}$ days. Therefore, for the facility to remain at capacity, we require that the inflow of newly incarcerated people equals the outflow of people released from confinement:

$$\rho_{in} C=\rho_{out}P,$$

where $C$ is the population size of the local community and $P$ is the population size of the correctional facility.

**Web Appendix 3: The Reproductive Numbers and the Probability of a Major Outbreak**

**The basic reproductive number.** To compute the basic reproductive number for COVID-19 infection we use standard next-generation techniques [[44]](https://paperpile.com/c/WghqcR/rc4q). Specifically, we use the stochastic model (Web Table 1-4), which has six compartments that contribute to transmission: $C_{I},C_{A},W_{I},W_{A},P_{I},$ and $P_{A}$, along with routines for robust numerical calculation of Jacobian matrices [8]. It follows that the basic reproductive number for local communities (Web Figure 3) is

$$R_{0}^{C}=\rho\left( {F|}_{DFE, \beta_{WP}=0}V^{-1} \right)$$

and the basic reproductive number within incarceration facilities [[40]](https://paperpile.com/c/WghqcR/YCQV) is

$$R_{0}^{P}=\rho\left( {F|}_{DFE, \beta_{CW}=0}V^{-1} \right)$$

where DFE stands for the disease-free equilibrium, and $\rho$ is the spectral radius operator.

**The probability of a major outbreak.** Given the definition of the basic reproductive numbers, the probability of a major outbreak in the correctional facility given a single infected incarcerated individual [[14]](https://paperpile.com/c/WghqcR/LyNj) is approximately

$$1-\frac{1}{R_{0}^{P}}.$$

Similarly, the probability of a major outbreak in the local community is

$$1-\frac{1}{R_{0}^{C}}.$$

**
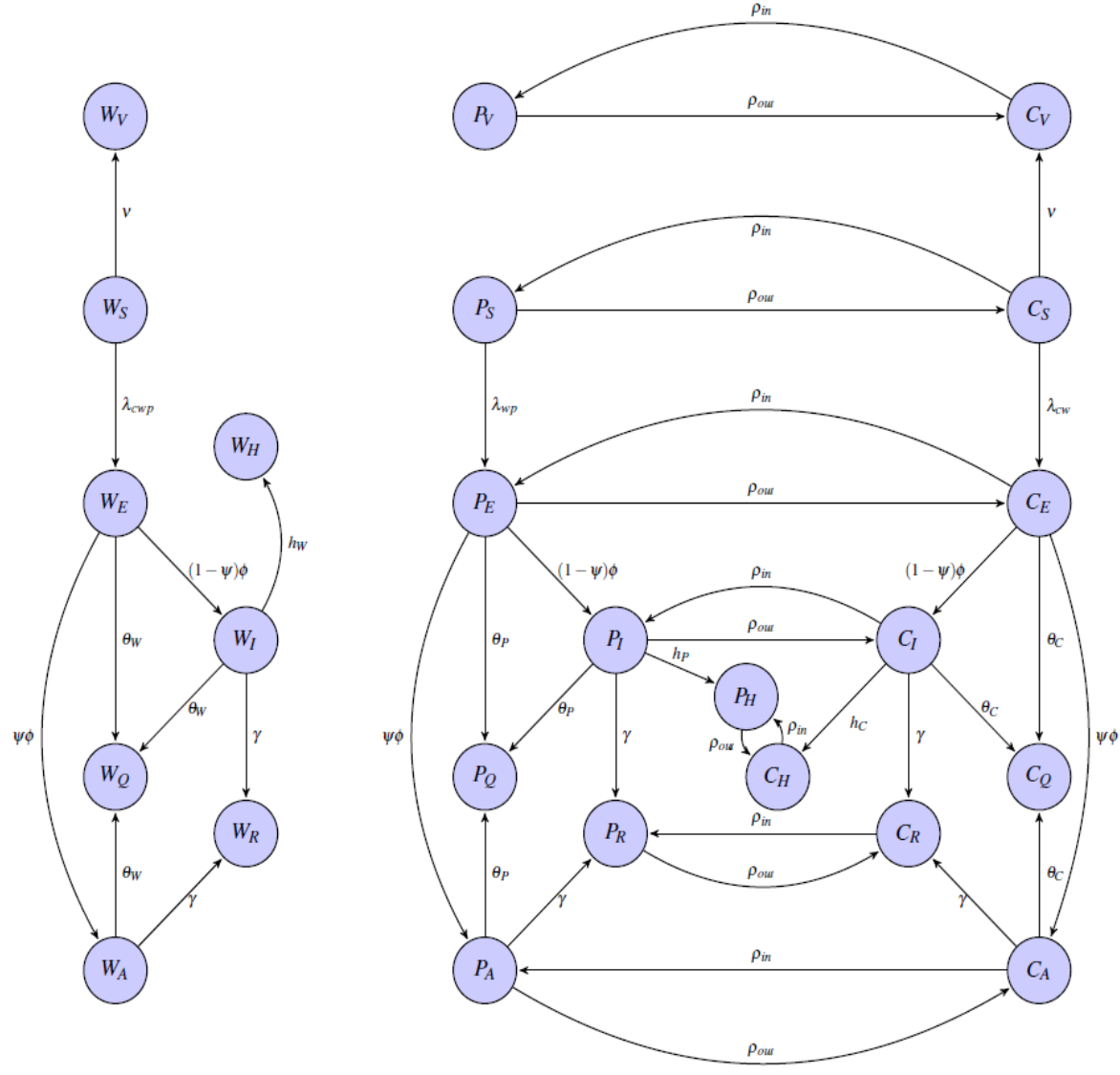
Web Figure 1.** Compartmental Diagram for COVID-19 Infection and Incarceration





Web Figure 2. Basic Reproductive Numbers. The basic reproductive number for COVID-19 in the local community (black dashed line) and inside the correctional facility (red solid) versus population size of the community.





Web Figure 3. Probability of a Major Outbreak in the Local Community. The probability of a major outbreak of COVID-19 in the local community, $1-\frac{1}{R_{0}^{C}}$, for the given community size. .

Web Table 1. **Transition Probabilities for COVID-19 Infection of Incarcerated Individuals.** Transitions indicate the compartments that are decreased and increased by 1 person.

| Event | Transition during $\Delta t$ | Transition probability $p_{j\leftarrow i} (\Delta t)$ |
| --- | --- | --- |
| Infection of a incarcerated person | $P_{s}\to P_{s}-1$  $P_{E}\to P_{E}+1$ | $\lambda_{CW}P_{S}\Delta t+o(\Delta t)$ |
| Latently infected incarcerated person  becomes asymptomatic | $P_{E}\to P_{E}-1$  $P_{A}\to P_{A}+1$ | $\psi{\phi P}_{E}\Delta t+o(\Delta t)$ |
| Latently infected incarcerated person  becomes symptomatic | $P_{E}\to P_{E}-1$  $P_{I}\to P_{I}+1$ | $\left( 1-\psi\right)\phi P_{E}\Delta t+o(\Delta t)$ |
| Latently infected incarcerated person  is quarantined | $P_{E}\to P_{E}-1$  $P_{Q}\to P_{Q}+1$ | ${\theta_{P}P}_{E}\Delta t+o(\Delta t)$ |
| Asymptomatic incarcerated person  recovers | $P_{A}\to P_{A}-1$  $P_{R}\to P_{R}+1$ | ${\gamma P}_{A}\Delta t+o(\Delta t)$ |
| Asymptomatic incarcerated person  is quarantined | $P_{A}\to P_{A}-1$  $P_{Q}\to P_{Q}+1$ | ${\theta_{P}P}_{A}\Delta t+o(\Delta t)$ |
| Symptomatic incarcerated person recovers | $P_{I}\to P_{I}-1$  $P_{R}\to P_{R}+1$ | ${\gamma P}_{I}\Delta t+o(\Delta t)$ |
| Symptomatic incarcerated person is quarantined | $P_{I}\to P_{I}-1$  $P_{Q}\to P_{Q}+1$ | ${\theta_{P}P}_{I}\Delta t+o(\Delta t)$ |
| Symptomatic incarcerated person is hospitalized | $P_{I}\to P_{I}-1$  $P_{H}\to P_{H}+1$ | ${h_{P}P}_{I}\Delta t+o(\Delta t)$ |
|  |  |  |
| Hospitalized incarcerated person dies | $P_{H}\to P_{H}-1$  $P_{D}\to P_{D}+1$ | ${\mu_{P}P}_{H}\Delta t+o(\Delta t)$ |

Web Table 2 **Transition Probabilities for COVID-19 Infection of** Correctional Workers. Transitions indicate the compartments that are decreased and increased by 1 person.

| Event | Transition during $\Delta t$ | Transition probability $p_{j\leftarrow i} (\Delta t)$ |
| --- | --- | --- |
| Infection of a correctional worker | $W_{s}\to W_{s}-1$  $W_{E}\to W_{E}+1$ | $\lambda_{CWP}W_{S}\Delta t+o(\Delta t)$ |
| Latently infected correctional worker becomes asymptomatic | $W_{E}\to W_{E}-1$  $W_{A}\to W_{A}+1$ | $\psi{\phi W}_{E}\Delta t+o(\Delta t)$ |
| Latently infected correctional worker becomes symptomatic | $W_{E}\to W_{E}-1$  $W_{I}\to W_{I}+1$ | $\left( 1-\psi\right)\phi W_{E}\Delta t+o(\Delta t)$ |
| Latently infected correctional worker is quarantined | $W_{E}\to W_{E}-1$  $W_{Q}\to W_{Q}+1$ | ${\theta_{c}W}_{E}\Delta t+o(\Delta t)$ |
| Asymptomatic correctional worker recovers | $W_{A}\to W_{A}-1$  $W_{R}\to W_{R}+1$ | ${\gamma W}_{A}\Delta t+o(\Delta t)$ |
| Asymptomatic correctional worker is quarantined | $W_{A}\to W_{A}-1$  $W_{Q}\to W_{Q}+1$ | ${\theta_{c}W}_{A}\Delta t+o(\Delta t)$ |
| Symptomatic correctional worker recovers | $W_{I}\to W_{I}-1$  $W_{R}\to W_{R}+1$ | ${\gamma W}_{I}\Delta t+o(\Delta t)$ |
| Symptomatic correctional worker is quarantined | $W_{I}\to W_{I}-1$  $W_{Q}\to W_{Q}+1$ | ${\theta_{c}W}_{I}\Delta t+o(\Delta t)$ |
| Symptomatic correctional worker is hospitalized | $W_{I}\to W_{I}-1$  $W_{H}\to W_{H}+1$ | ${h_{W}W}_{I}\Delta t+o(\Delta t)$ |
| Susceptible correctional worker is vaccinated | $W_{S}\to W_{S}-1$  $W_{V}\to W_{V}+1$ | ${\nu W}_{S}\Delta t+o(\Delta t)$ |
| Hospitalized correctional worker dies | $W_{H}\to W_{H}-1$  $W_{D}\to W_{D}+1$ | $\mu_{C}W_{H}\Delta t+o(\Delta t)$ |

Web Table 3. **Transition Probabilities for COVID-19 Infection of** the Local Community. Transitions indicate the compartments that are decreased and increased by 1 person.

| Event | Transition during $\Delta t$ | Transition probability $p_{j\leftarrow i} (\Delta t)$ |
| --- | --- | --- |
| Infection of a community member | $C_{s}\to C_{s}-1$  $C_{E}\to C_{E}+1$ | $\lambda_{CW}C_{S}\Delta t+o(\Delta t)$ |
| Latently infected community member becomes asymptomatic | $C_{E}\to C_{E}-1$  $C_{A}\to C_{A}+1$ | $\psi{\phi C}_{E}\Delta t+o(\Delta t)$ |
| Latently infected community member becomes symptomatic | $C_{E}\to C_{E}-1$  $C_{I}\to C_{I}+1$ | $\left( 1-\psi\right)\phi C_{E}\Delta t+o(\Delta t)$ |
| Latently infected community member is quarantined | $C_{E}\to C_{E}-1$  $C_{Q}\to C_{Q}+1$ | ${\theta_{c}C}_{E}\Delta t+o(\Delta t)$ |
| Asymptomatic community member recovers | $C_{A}\to C_{A}-1$  $C_{R}\to C_{R}+1$ | ${\gamma C}_{A}\Delta t+o(\Delta t)$ |
| Asymptomatic community member is quarantined | $C_{A}\to C_{A}-1$  $C_{Q}\to C_{Q}+1$ | ${\theta_{c}C}_{A}\Delta t+o(\Delta t)$ |
| Symptomatic community member recovers | $C_{I}\to C_{I}-1$  $C_{R}\to C_{R}+1$ | ${\gamma C}_{I}\Delta t+o(\Delta t)$ |
| Symptomatic community member is quarantined | $C_{I}\to C_{I}-1$  $C_{Q}\to C_{Q}+1$ | ${\theta_{c}C}_{I}\Delta t+o(\Delta t)$ |
| Symptomatic community member is hospitalized | $C_{I}\to C_{I}-1$  $C_{H}\to C_{H}+1$ | ${h_{c}C}_{I}\Delta t+o(\Delta t)$ |
| Susceptible community member is vaccinated | $C_{S}\to C_{S}-1$  $C_{V}\to C_{V}+1$ | ${\nu C}_{S}\Delta t+o(\Delta t)$ |
| Hospitalized community member dies | $C_{H}\to C_{H}-1$  $C_{D}\to C_{D}+1$ | ${h_{C}C}_{H}\Delta t+o(\Delta t)$ |

Web Table 4. **Transition Probabilities for the Incarceration of Local Community Members and the Release of Incarcerated Individuals.** Transitions indicate the compartments that are decreased and increased by 1 person.

| Event | Transition during $\Delta t$ | Transition probability $p_{j\leftarrow i} (\Delta t)$ |
| --- | --- | --- |
| Susceptible community member is incarcerated | $C_{s}\to C_{s}-1$  $P_{s}\to P_{s}+1$ | $\rho_{in}C_{s}\Delta t+o(\Delta t)$ |
| Latently infected community member is incarcerated | $C_{E}\to C_{s}-1$  $P_{E}\to P_{E}+1$ | $\rho_{in}C_{E}\Delta t+o(\Delta t)$ |
| Symptomatic community member is incarcerated | $C_{I}\to C_{I}-1$  $P_{I}\to P_{I}+1$ | $\rho_{in}C_{I} \Delta t+o(\Delta t)$ |
| Asymptomatic community member is incarcerated | $C_{A}\to C_{A}-1$  $P_{A}\to P_{A}+1$ | $\rho_{in}C_{A}\Delta t+o(\Delta t)$ |
| Recovered community member is incarcerated | $C_{R}\to C_{R}-1$  $P_{R}\to P_{R}+1$ | $\rho_{in}C_{R}\Delta t+o(\Delta t)$ |
| Quarantined community member is incarcerated | $C_{Q}\to C_{Q}-1$  $P_{Q}\to P_{Q}+1$ | $\rho_{in}C_{Q}\Delta t+o(\Delta t)$ |
| Susceptible incarcerated person is released | $C_{s}\to C_{s}+1$  $P_{s}\to P_{s}-1$ | $\rho_{out}P_{s}\Delta t+o(\Delta t)$ |
| Latently infected incarcerated person is released | $C_{E}\to C_{E}+1$  $P_{E}\to P_{E}-1$ | $\rho_{out}P_{E}\Delta t+o(\Delta t)$ |
| Symptomatic incarcerated person is released | $C_{I}\to C_{I}+1$  $P_{I}\to P_{I}-1$ | $\rho_{out}P_{I}\Delta t+o(\Delta t)$ |
| Asymptomatic incarcerated person is released | $C_{A}\to C_{A}+1$  $P_{A}\to P_{A}-1$ | $\rho_{out}P_{A}\Delta t+o(\Delta t)$ |
| Recovered incarcerated person is released | $C_{R}\to C_{R}+1$  $P_{R}\to P_{R}-1$ | $\rho_{out}P_{R}\Delta t+o(\Delta t)$ |
| Quarantined incarcerated person is released | $C_{Q}\to C_{Q}+1$  $P_{Q}\to P_{Q}-1$ | $\rho_{out}P_{Q}\Delta t+o(\Delta t)$ |

Web Table 5. Parameter Names, Values, and Sources.

| Definition | Parameter | Point estimate | Reference |
| --- | --- | --- | --- |
| Population size |  |  |  |
| Total | $N$ | 5,000 10,000 20,000 |  |
| Local community members | $C_{tot}$ | 3,780  8,780  18,780 |  |
| Correctional workers | $W_{tot}$ | 420 | [[7]](https://paperpile.com/c/WghqcR/zOTV) |
| Incarcerated people | $P_{tot}$ | 800 | [[7]](https://paperpile.com/c/WghqcR/zOTV) |
| Basic reproductive number | ${R_{0}}^{C}$ | 1.91-2.45 | ${}^{a}$ |
| Transmission rate |  |  |  |
| Community | $\beta_{CW}$ | 0.865$day^{-1}$ | [[37]](https://paperpile.com/c/WghqcR/y9LU) |
| Correctional facility | $\beta_{WP}$ | $1.0383 day^{-1}$ | ${}^{a}$ |
| Latent period | $1/ \phi$ | 5.1 days | [[37]](https://paperpile.com/c/WghqcR/y9LU) |
| Infectious period | $1/ \gamma$ | 3.7 days | [[39]](https://paperpile.com/c/WghqcR/iKcL) |
| Incarceration rate | $\rho_{in}$ | $\rho_{out}\frac{P}{C}$ |  |
| Incarceration period | $1/\rho_{out}$ |  |  |
| Jail |  | 25 days | [[45]](https://paperpile.com/c/WghqcR/9DLp) |
| State Prison |  | 2.6 years | [[4]](https://paperpile.com/c/WghqcR/6Kfa) |
| Proportion of incidence that are asymptomatic | $\psi$ | 0.179 | [[46]](https://paperpile.com/c/WghqcR/gDaU) |
| Vaccination rate | $\nu(t)$ |  | ${}^{a}$ |
| Hospitalization rate |  |  |  |
| General community | $h_{C}$ | 0.018 $day^{-1}$ | [[16]](https://paperpile.com/c/WghqcR/GLnu) |
| Incarceration workers | $h_{W}$ | 0.018 $day^{-1}$ | [[16]](https://paperpile.com/c/WghqcR/GLnu) |
| Incarcerated people | $h_{P}$ | 0.018 $day^{-1}$ | [[16]](https://paperpile.com/c/WghqcR/GLnu) |
| Mortality rate |  |  |  |
| Local community members | $\mu_{C}$ | 0.0015 $day^{-1}$ | [[16]](https://paperpile.com/c/WghqcR/GLnu) |
| Correctional workers | $\mu_{W}$ | 0.0015 $day^{-1}$ | [[16]](https://paperpile.com/c/WghqcR/GLnu) |
| Incarcerated people | $\mu_{P}$ | $\frac{39}{29}\mu_{C}$ | [[1]](https://paperpile.com/c/WghqcR/oVhr) |
| Quarantine rate |  |  |  |
| Local community members | $\theta_{C}$ | 0.116 $day^{-1}$ | [[37]](https://paperpile.com/c/WghqcR/y9LU) |
| Correctional workers | $\theta_{W}$ | 0.116 $day^{-1}$ | [[37]](https://paperpile.com/c/WghqcR/y9LU) |
| Incarcerated people | $\theta_{P}$ | $\xi\theta_{C}$ |  |
| Effect of intervention | $\xi$ | $\xi=0, 0.5, or 1.0$ |  |
| COVID-19 DALY weights |  |  |  |
| Moderate symptoms | $D_{M}$ | 0.051 | [[11]](https://paperpile.com/c/WghqcR/ur5u) |
| Severe symptoms | $D_{S}$ | 0.133 | [[11]](https://paperpile.com/c/WghqcR/ur5u) |
| Critical symptoms | $D_{C}$ | 0.655 | [[11]](https://paperpile.com/c/WghqcR/ur5u) |
| Death | $D_{D}$ | 1.000 | [[11]](https://paperpile.com/c/WghqcR/ur5u) |
| Frequency of COVID symptoms |  |  |  |
| Moderate symptoms |  | 0.784 | [[12]](https://paperpile.com/c/WghqcR/Cr03) |
| Severe symptoms |  | 0.138 | [[12]](https://paperpile.com/c/WghqcR/Cr03) |
| Critical symptoms |  | 0.061 | [[12]](https://paperpile.com/c/WghqcR/Cr03) |
| Duration of COVID-19 symptom type |  |  |  |
| Moderate symptoms |  | 1.5 weeks | [[47]](https://paperpile.com/c/WghqcR/rsk0) |
| Severe symptoms |  | 6 weeks | [[47]](https://paperpile.com/c/WghqcR/rsk0) |
| Critical symptoms |  | 6 weeks | [[47]](https://paperpile.com/c/WghqcR/rsk0) |
| Life expectancy at age of death due to COVID-19 | $L$ | 1 year | [[48]](https://paperpile.com/c/WghqcR/rsk0) |
| DALY discount rate | $r$ | 0.03 | [[49]](https://paperpile.com/c/WghqcR/Rjwd) |

${}^{a}$- Details available in Web Materials Parameter Estimation.


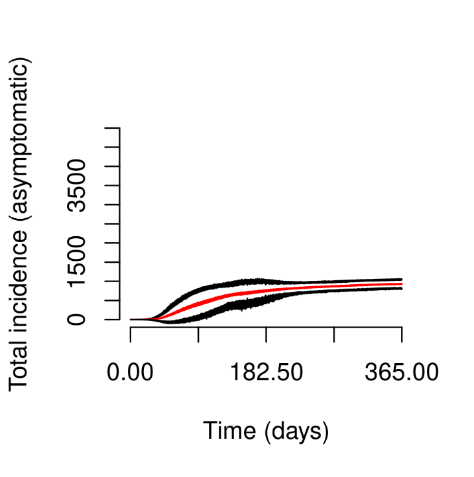

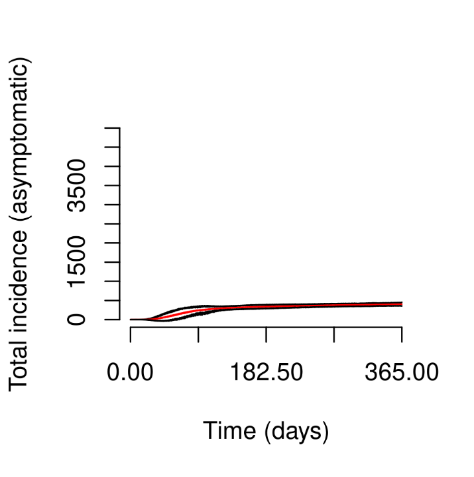

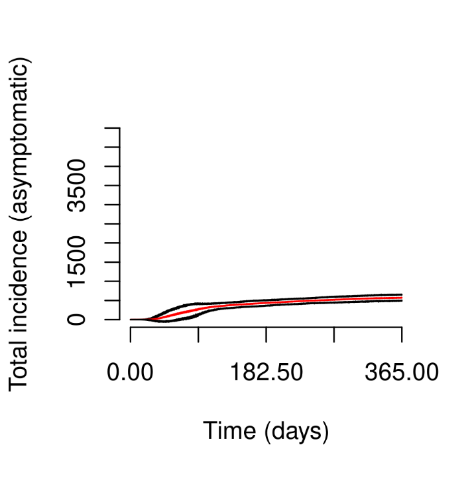

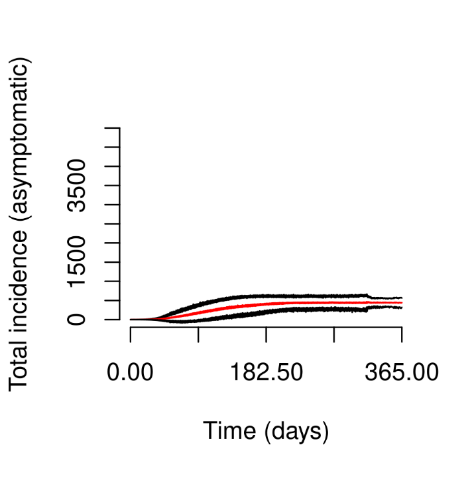

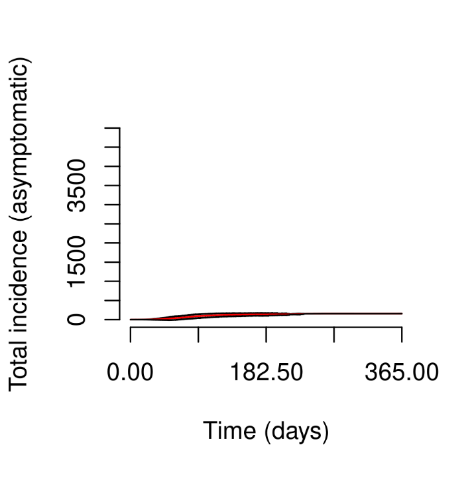

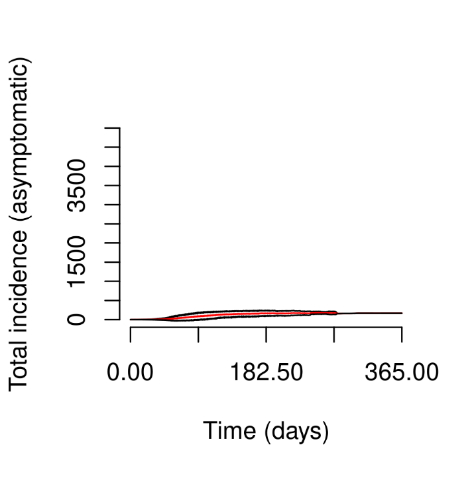

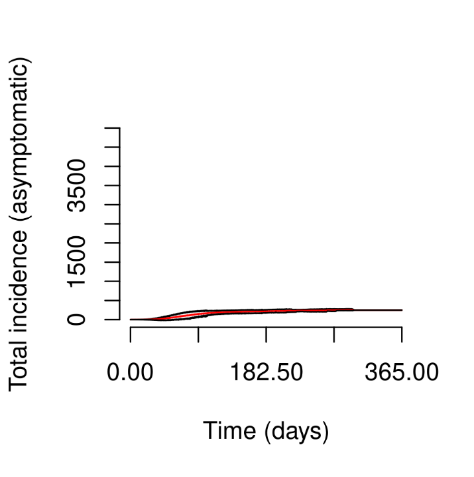

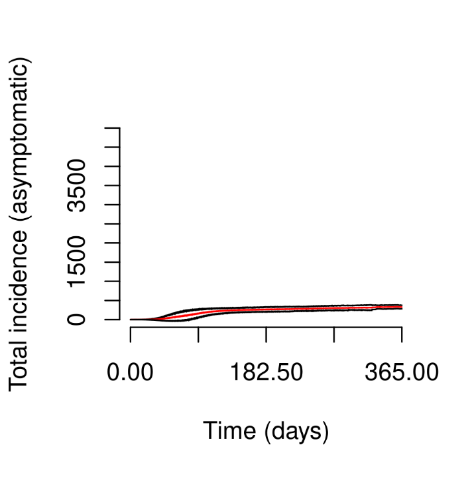

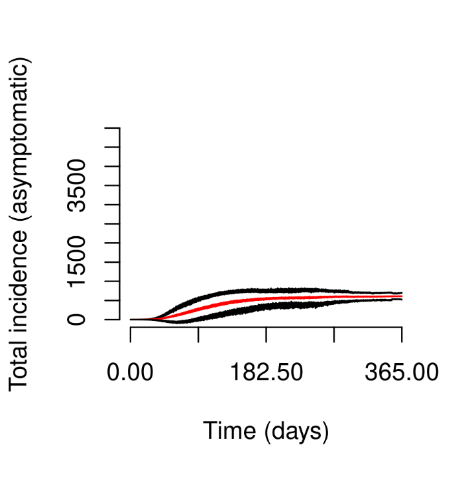


**Web Figure 4.** Asymptomatic COVID-19 infections for communities with correctional facilities with an average duration of incarceration of 25 days. Predictions correspond to the average of 10,000 stochastic realizations and 95% CI (black) for the entire population (top), the community (middle), and in the correctional facility (bottom) for population sizes of 5000 people (left), 10000 people (center), and 20000 people (right).


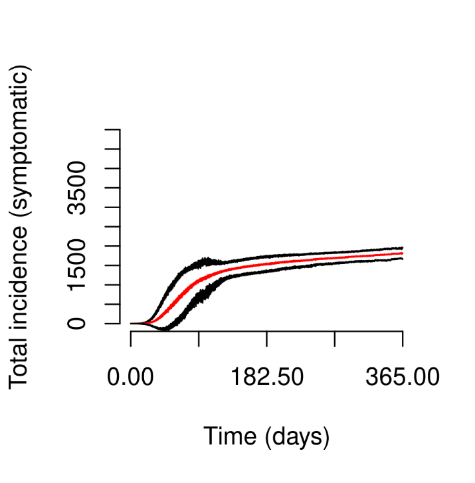

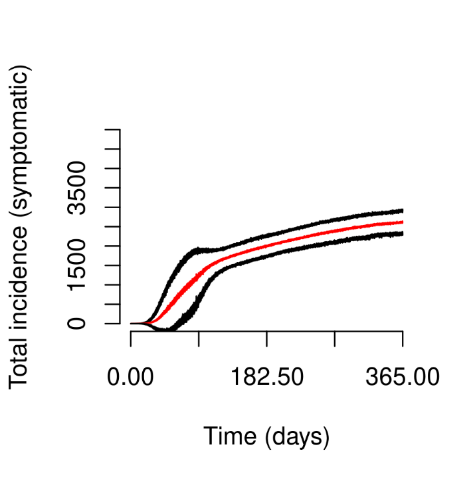

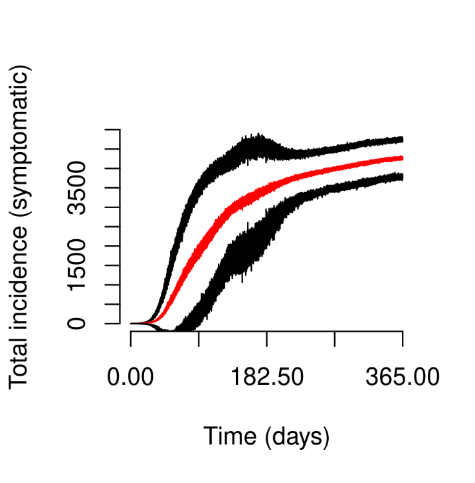

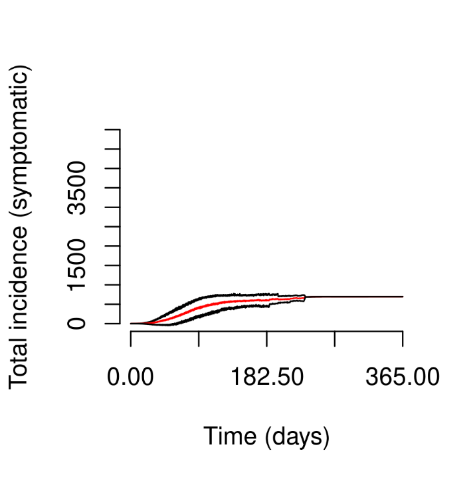

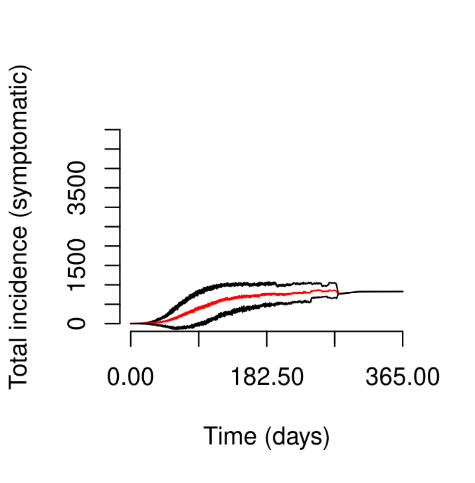

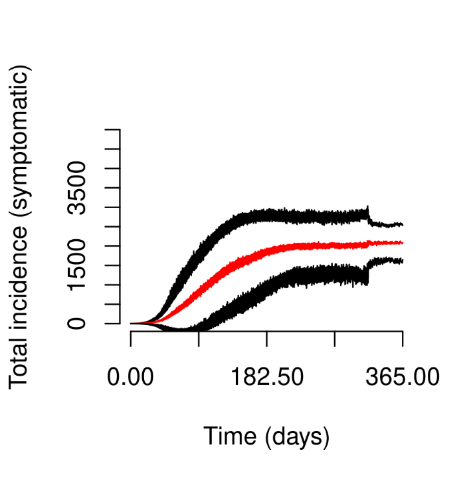

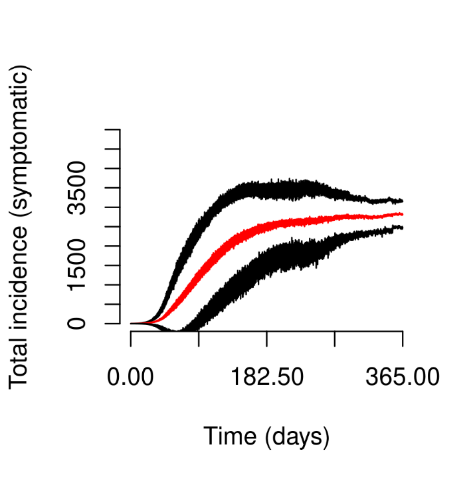

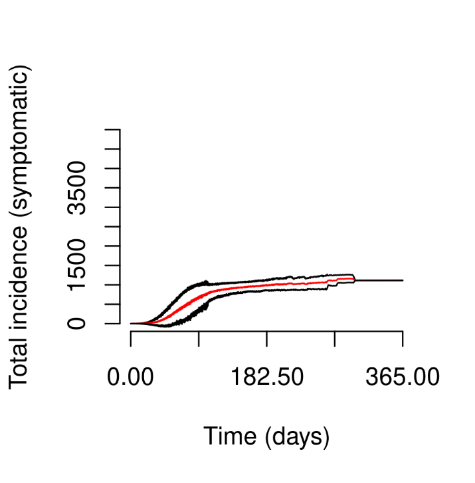

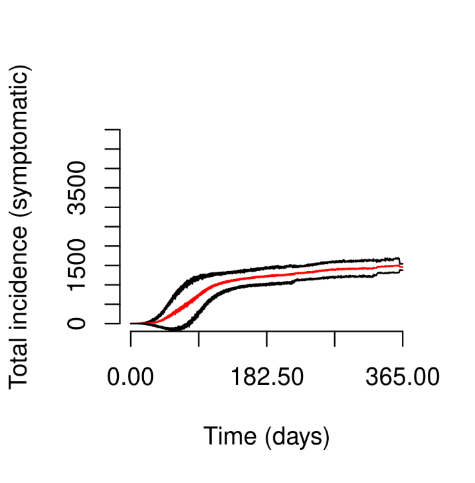


**Web Figure 5.** Symptomatic COVID-19 infections for communities with correctional facilities with an average duration of incarceration of 25 days. Predictions correspond to the average of 10,000 stochastic realizations and 95% CI (black) for the entire population (top), the community (middle), and in the correctional facility (bottom) for population sizes of 5000 people (left), 10000 people (center), and 20000 people (right).


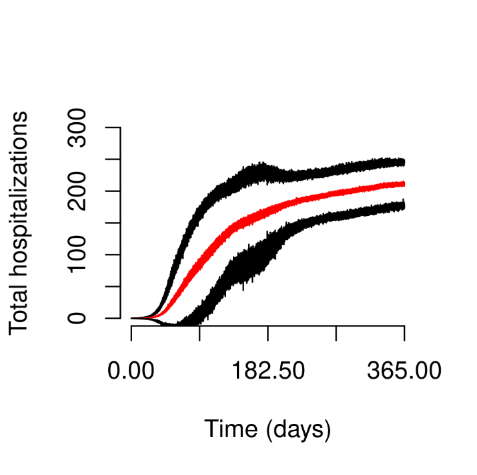

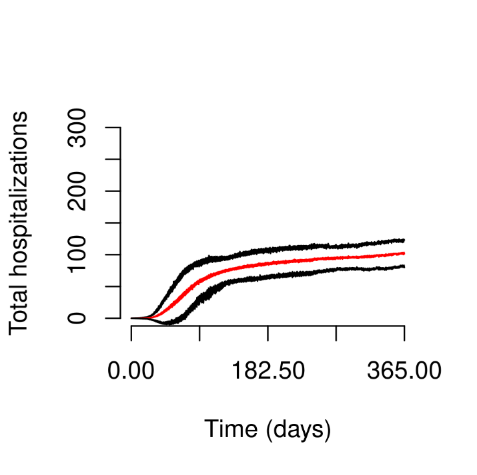

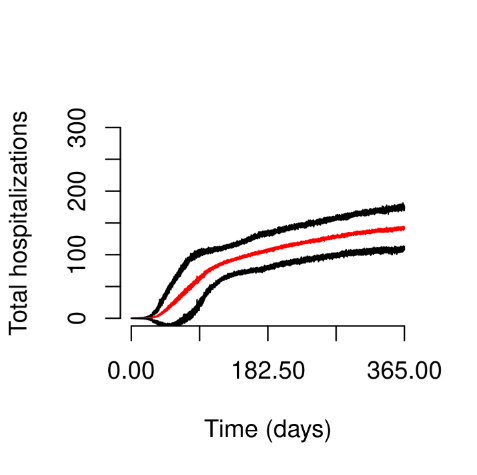

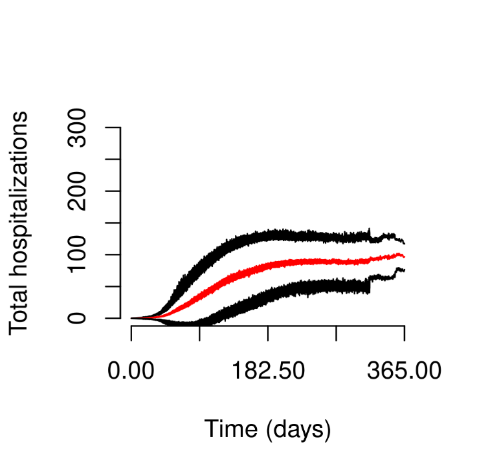

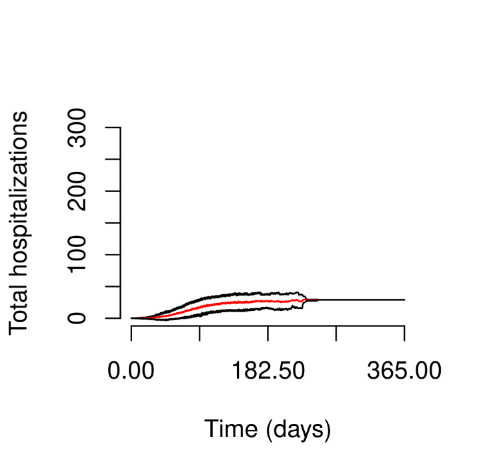

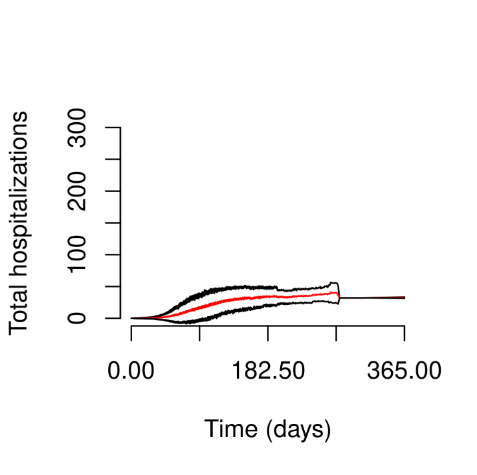

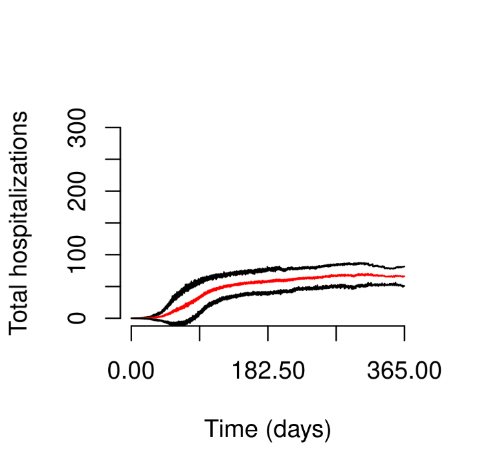

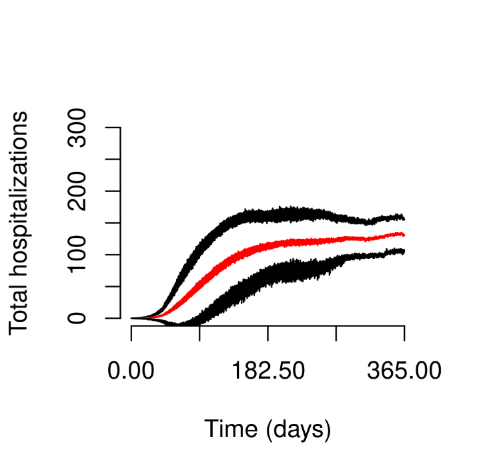

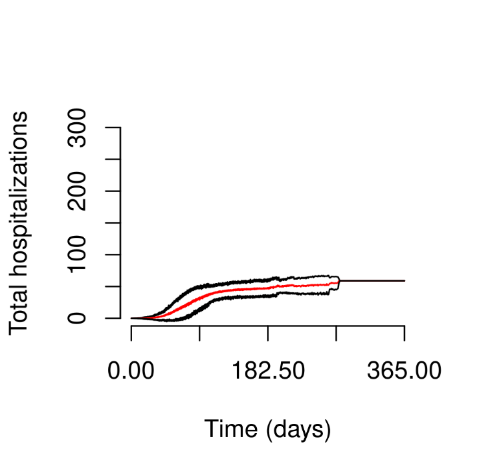


**Web Figure 6.** Hospitalized COVID-19 infections for communities with correctional facilities with an average duration of incarceration of 25 days. Predictions correspond to the average of 10,000 stochastic realizations and 95% CI (black) for the entire population (top), the community (middle), and in the correctional facility (bottom) for population sizes of 5000 people (left), 10000 people (center), and 20000 people (right).


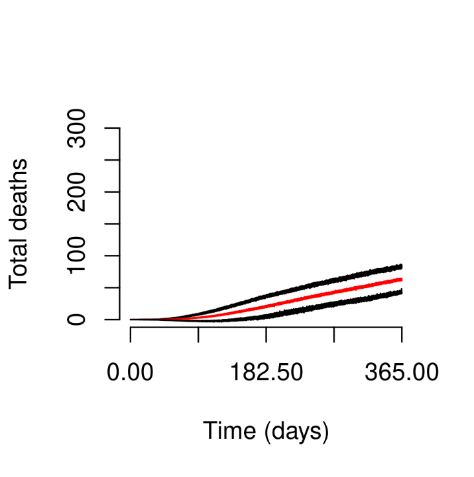

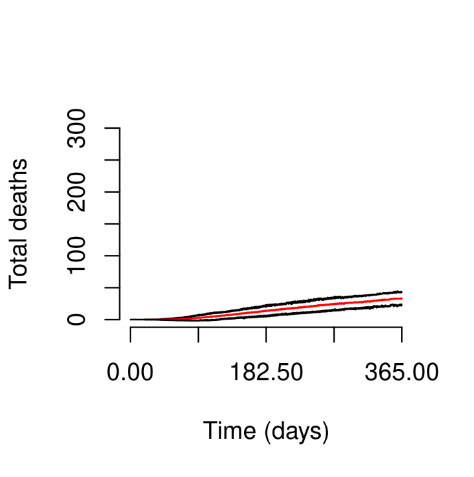

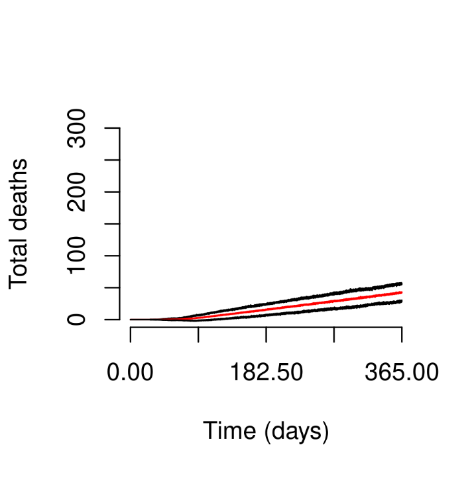

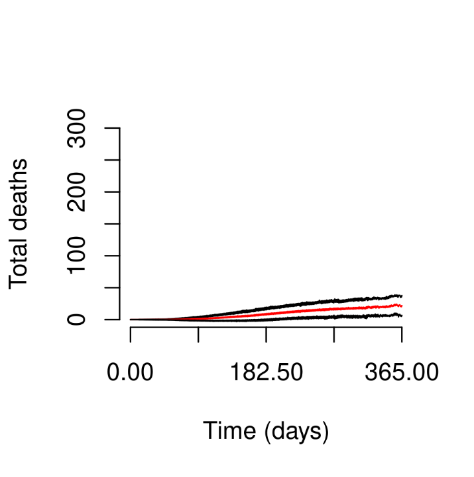

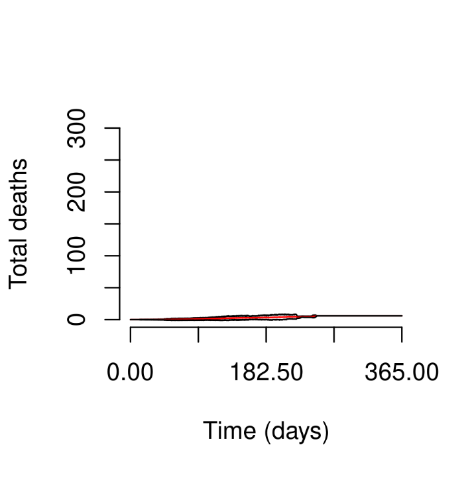

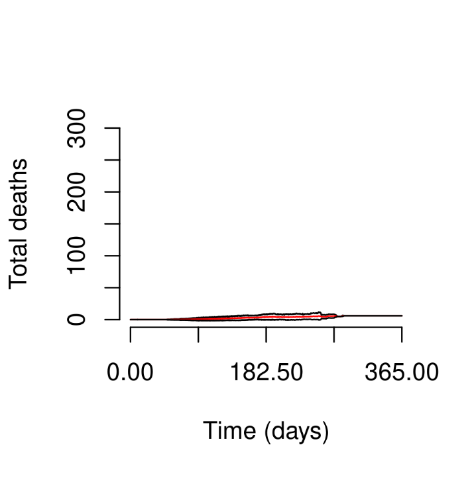

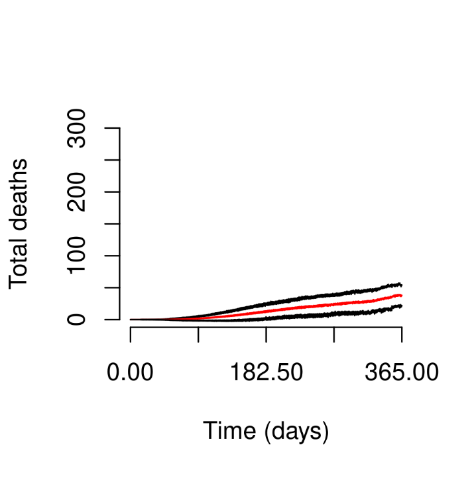

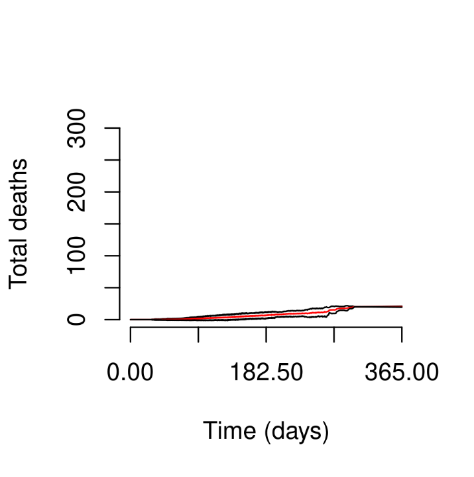

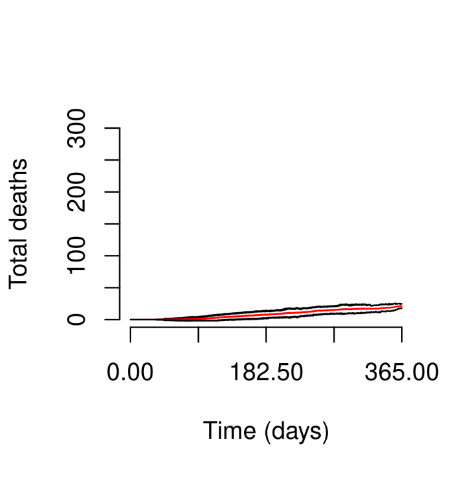


**Web Figure 7.** Deaths due to COVID-19 infections for communities with correctional facilities with an average duration of incarceration of 25 days. Predictions correspond to the average of 10,000 stochastic realizations and 95% CI (black) for the entire population (top), the community (middle), and in the correctional facility (bottom) for population sizes of 5000 people (left), 10000 people (center), and 20000 people (right).


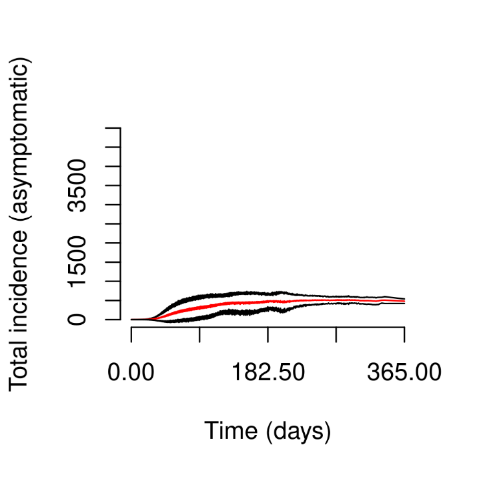

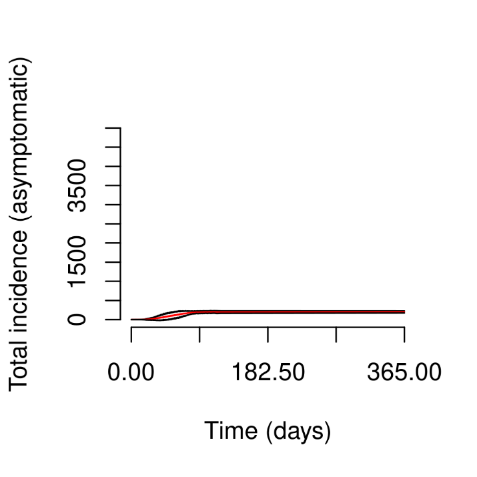

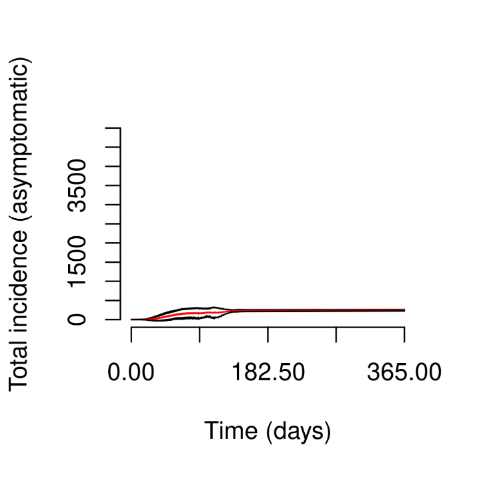

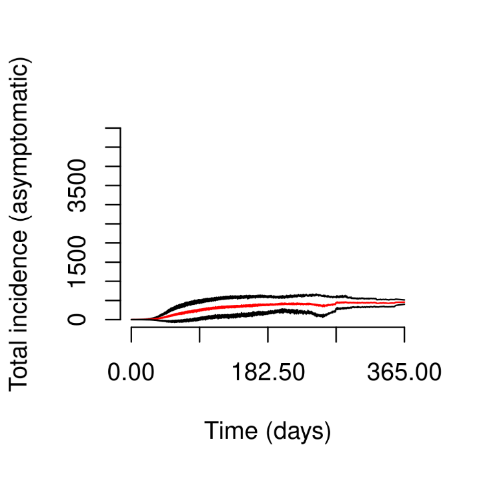

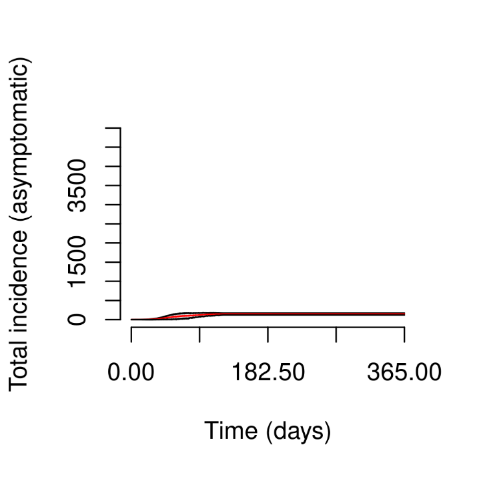

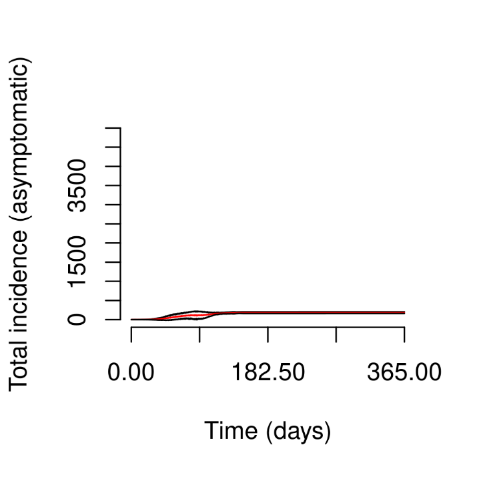

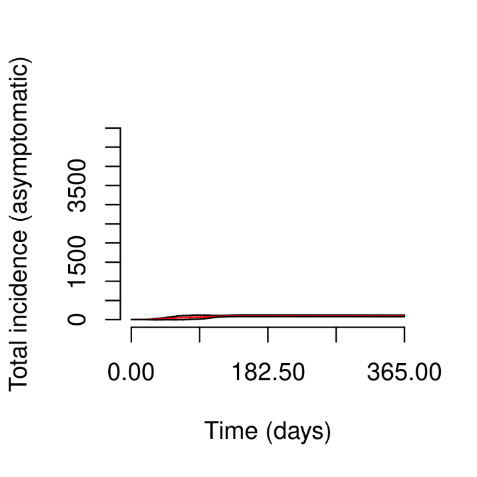

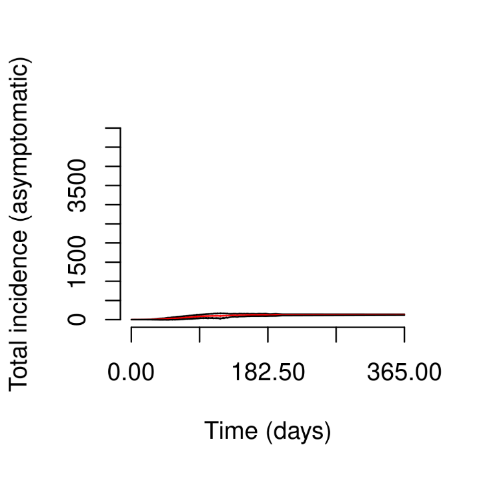

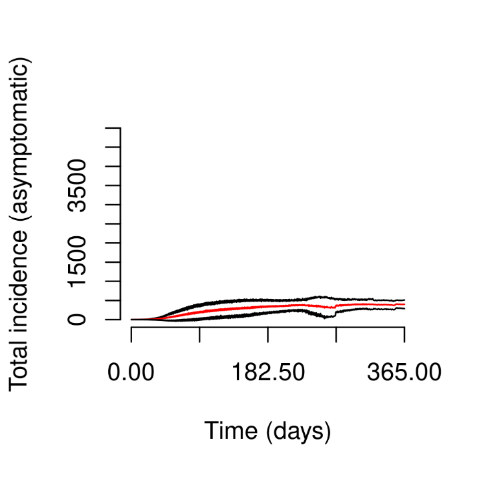


**Web Figure 8.** Asymptomatic COVID-19 infections for communities with correctional facilities with an average duration of incarceration of 2.6 years. Predictions correspond to the average of 10,000 stochastic realizations and 95% CI (black) for the entire population (top), the community (middle), and in the correctional facility (bottom) for population sizes of 5000 people (left), 10000 people (center), and 20000 people (right).


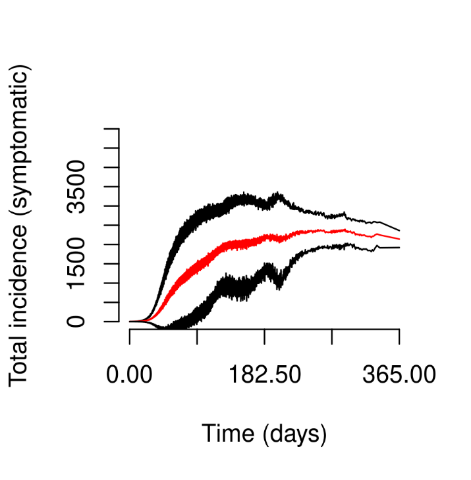

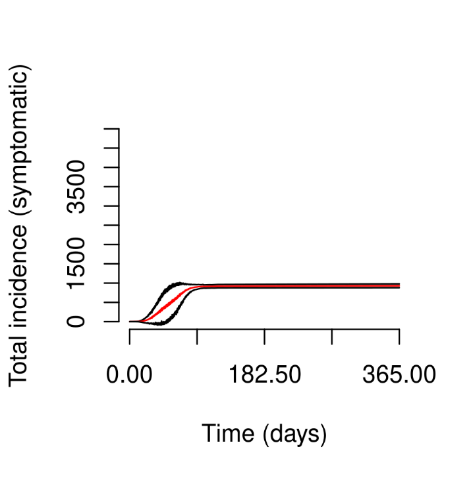

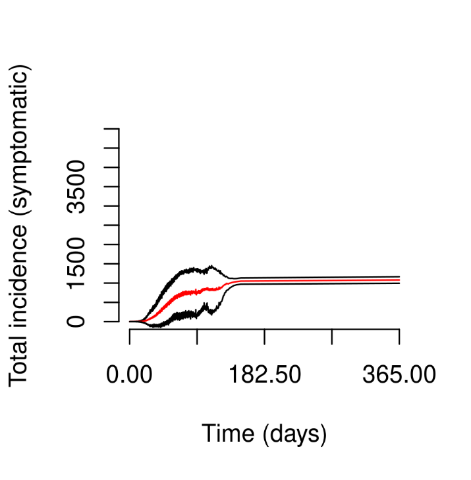

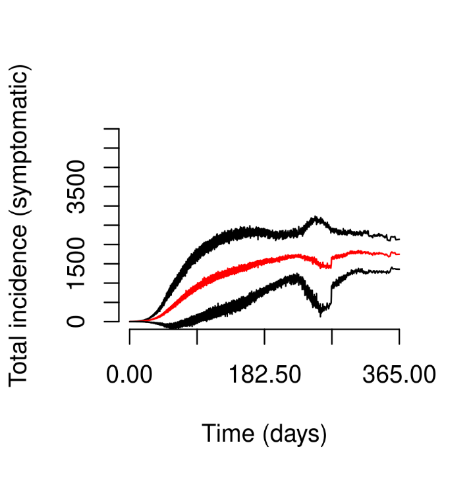

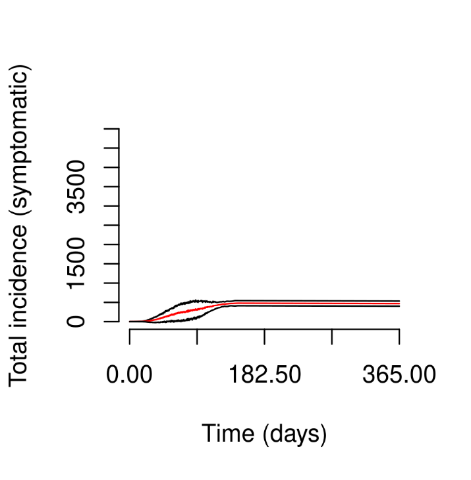

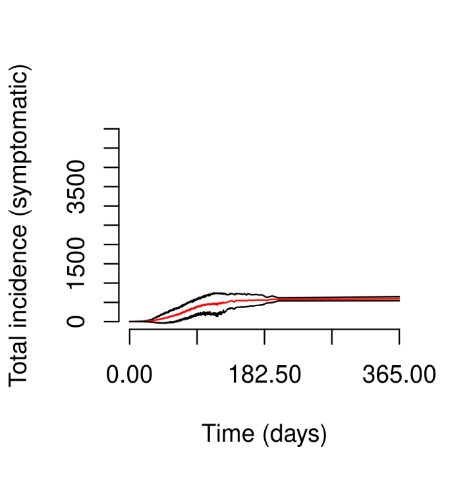

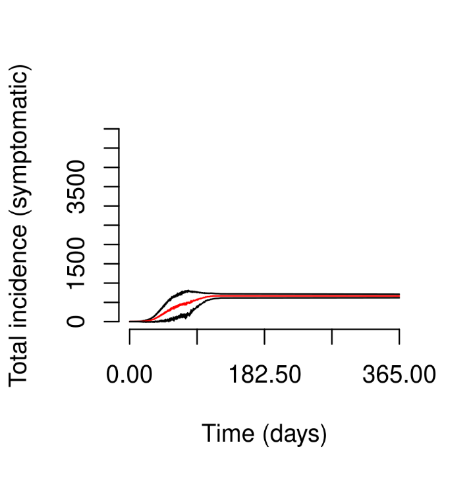

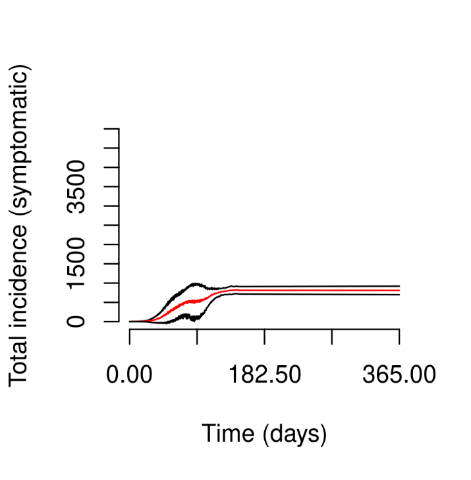

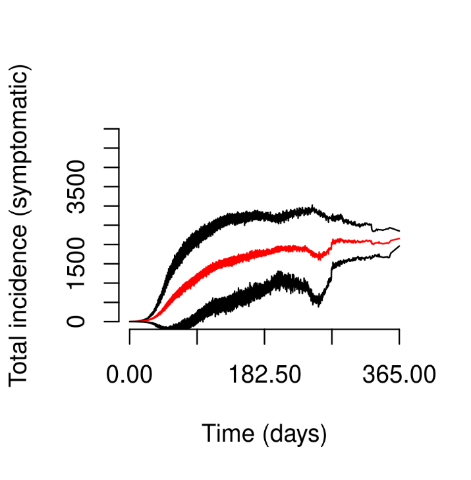


**Web Figure 9.** Symptomatic COVID-19 infections for communities with correctional facilities with an average duration of incarceration of 2.6 years. Predictions correspond to the average of 10,000 stochastic realizations and 95% CI (black) for the entire population (top), the community (middle), and in the correctional facility (bottom) for population sizes of 5000 people (left), 10000 people (center), and 20000 people (right).


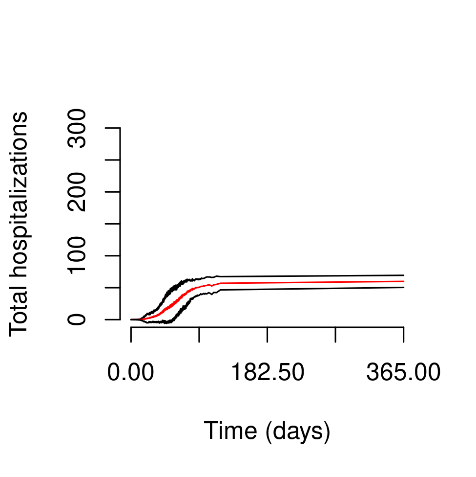

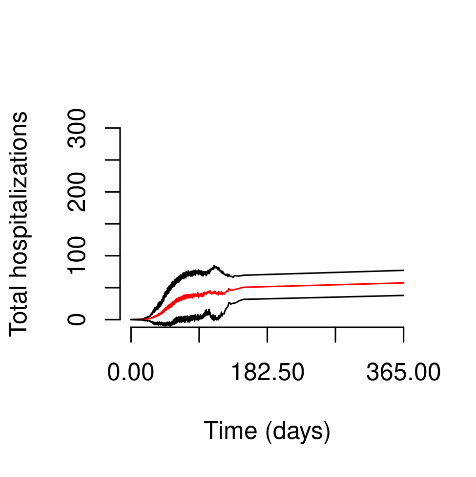

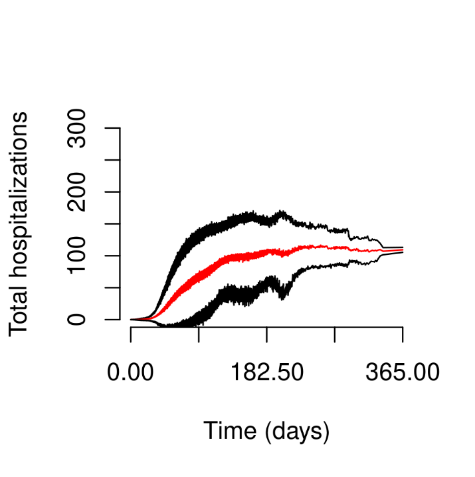

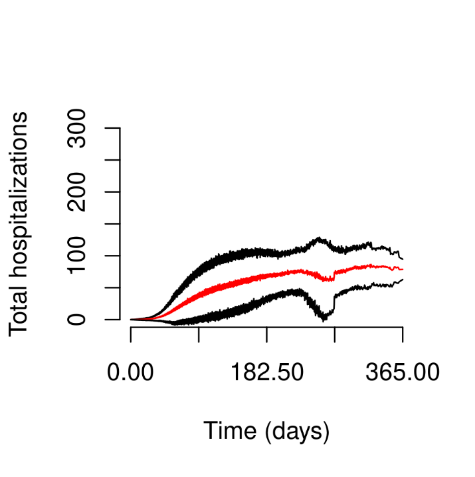

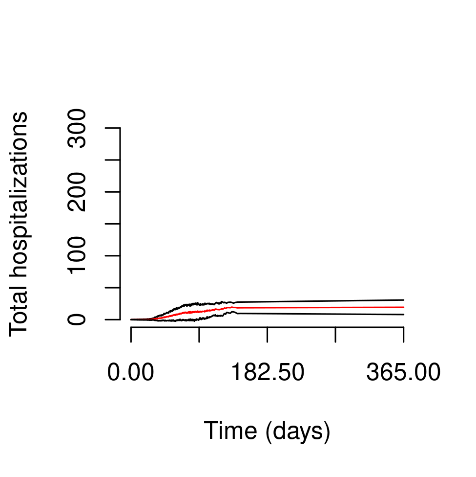

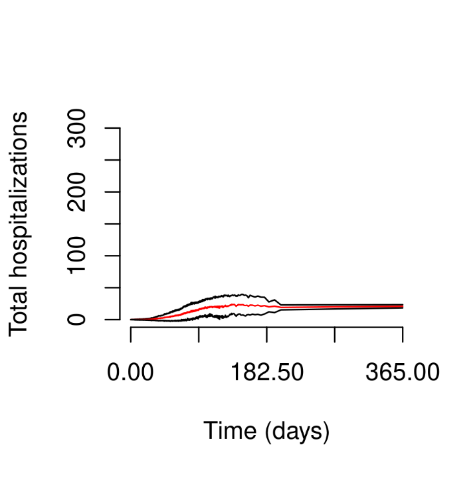

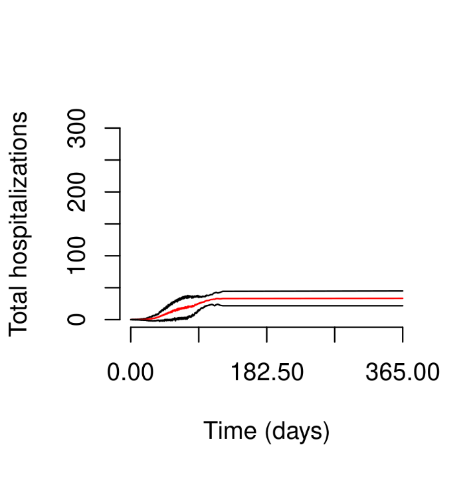

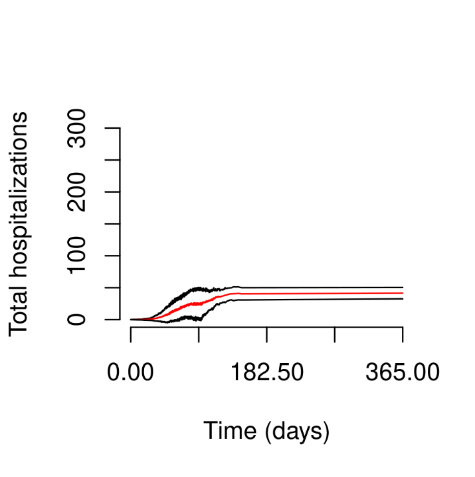

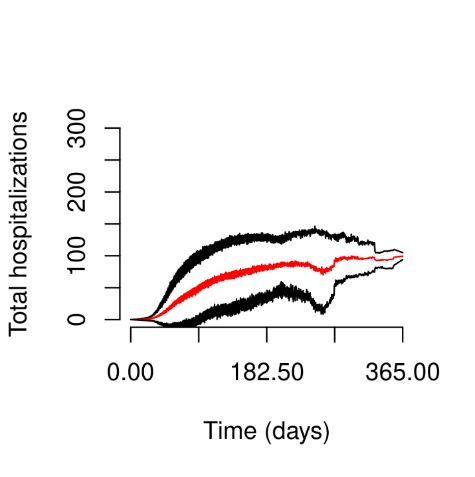


**Web Figure 10.** Hospitalized COVID-19 infections for communities with correctional facilities with an average duration of incarceration of 2.6 years. Predictions correspond to the average of 10,000 stochastic realizations and 95% CI (black) for the entire population (top), the community (middle), and in the correctional facility (bottom) for population sizes of 5000 people (left), 10000 people (center), and 20000 people (right).


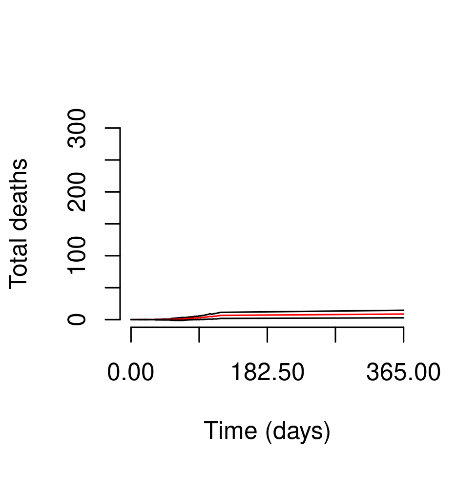

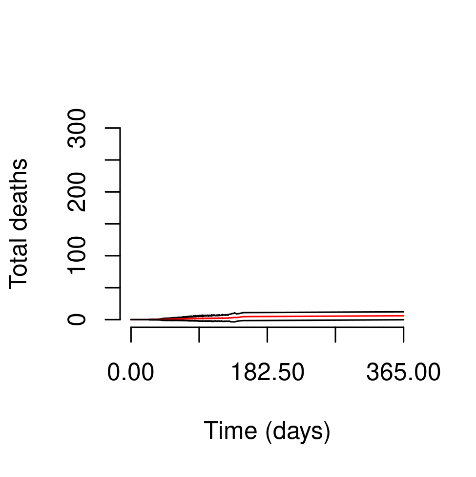

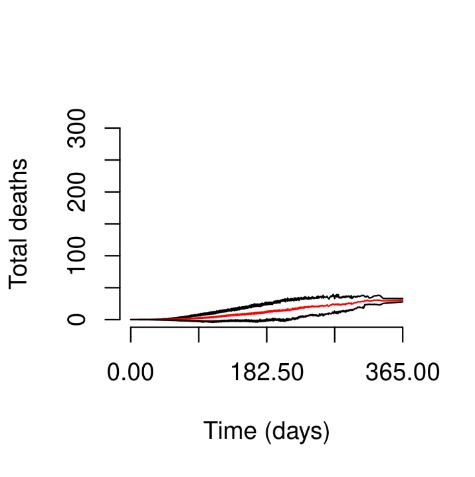

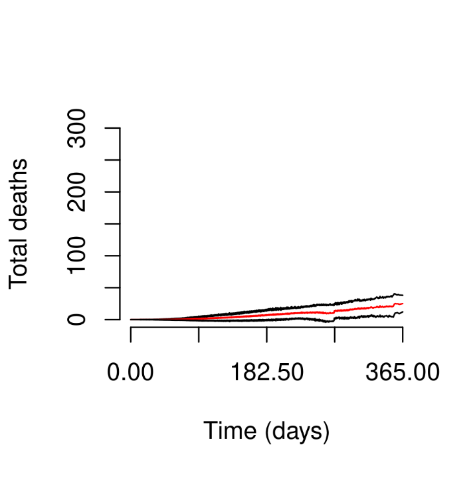

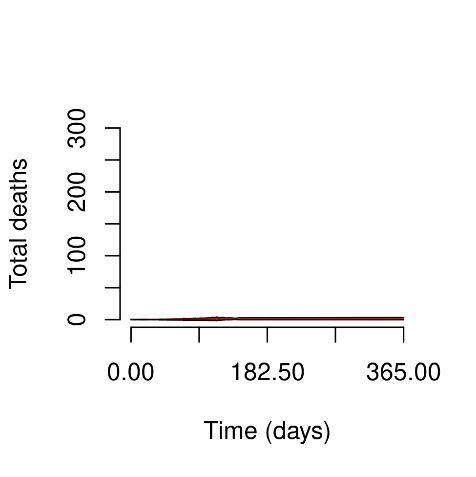

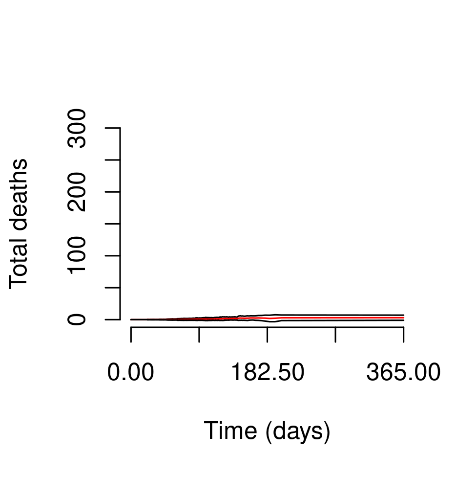

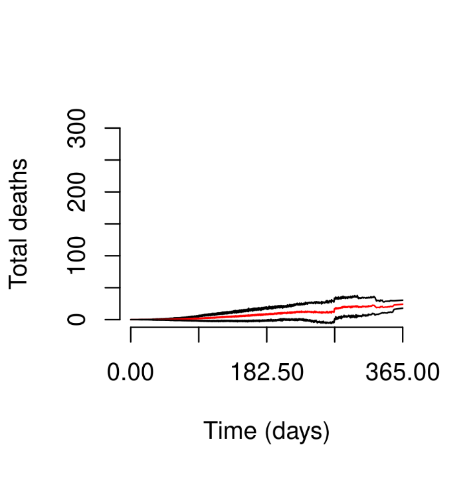

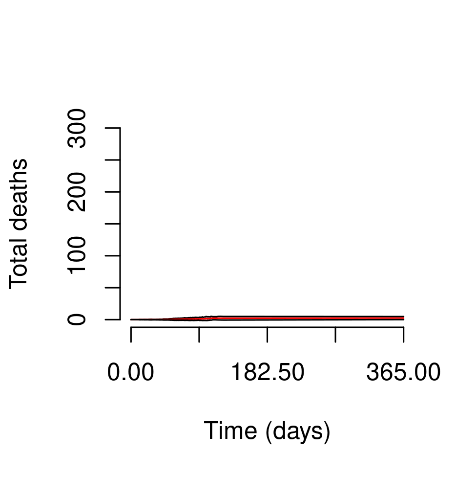

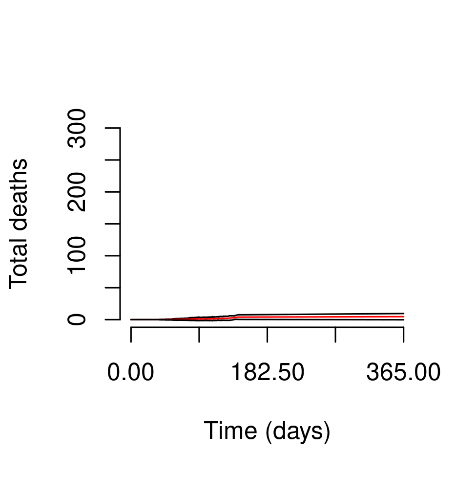


**Web Figure 11.** Deaths due to COVID-19 infections for communities with correctional facilities with an average duration of incarceration of 2.6 years. Predictions correspond to the average of 10,000 stochastic realizations and 95% CI (black) for the entire population (top), the community (middle), and in the correctional facility (bottom) for population sizes of 5000 people (left), 10000 people (center), and 20000 people (right).
